# Risk stratification for MDR/RR-TB: a comprehensive integrated patient-level analysis from TB programs and contemporary clinical trials

**DOI:** 10.1038/s41467-026-77931-0

**Published:** 2026-09-22

**Authors:** Maria Garcia-Cremades, Jonathon R. Campbell, Natasha Strydom, Belén P. Solans, Samer Mouksassi, Ziran Li, Patrick P. J. Phillips, Gustavo E. Velásquez, Payam Nahid, Dick Menzies, Radojka M. Savic

**Affiliations:** 1 Department of Bioengineering and Therapeutic Sciences, University of California, San Francisco, San Francisco, CA USA; 2 Department of Pharmaceutics and Food Technology, School of Pharmacy, Complutense University of Madrid, Madrid, Spain; 3 Institute of Industrial Pharmacy, Complutense University of Madrid, Madrid, Spain; 4 Department of Global and Public Health, McGill University, Montreal, QC Canada; 5 Respiratory Epidemiology and Clinical Research Unit, Centre for Outcomes Research and Evaluation, Research Institute of the McGill University Health Centre, Montreal, QC Canada; 6 Center for Tuberculosis, UCSF Institute for Global Health Sciences, University of California, San Francisco, San Francisco, CA USA; 7 Division of Pulmonary and Critical Care Medicine, University of California, San Francisco, San Francisco, CA USA; 8 Division of HIV, Infectious Diseases, and Global Medicine, University of California, San Francisco, San Francisco, CA USA; 9 Department of Medicine, McGill University, Montreal, QC Canada

**Keywords:** Tuberculosis, Risk factors

## Abstract

Tuberculosis is the deadliest infectious disease, and treatments for multidrug-resistant tuberculosis are lengthy and toxic. By analyzing a large international database (N = 7,750), we identified patient risk phenotypes linked to unfavorable outcomes. Factors like HIV infection, age, low BMI, smoking, acid-fast bacillus smear positivity, extrapulmonary involvement, and cavitary disease were associated (*p* < 0.001) with worse clinical outcomes (ROC AUC 0.728; 95% CI 0.726–0.731) and were used to derive a patient risk score. Low-risk patients had a lower probability of unfavorable outcome over time since treatment initiation compared to high-risk patients (*p* < 0.001). External validation with a highly effective 6-month BPaL regimen showed 97% treatment success for low-risk patients and 88% for medium-to-high-risk patients. Stratifying patients by risk phenotype enables more effective, personalized tuberculosis interventions, which are key to improving cure rates.

## Abstract (web summary)

Pooling data from 7750 patients with drug-resistant tuberculosis worldwide, in this study, the authors defined disease-severity phenotypes based on clinical and treatment features, stratifying patients by risk of unfavourable outcomes to enable risk-adapted, stratified care.

## Introduction

Multidrug-resistant or rifampicin-resistant tuberculosis (MDR/RR-TB) is caused by bacteria that are resistant to rifampicin (RR), or to at least isoniazid and rifampin (MDR), two of the most potent TB drugs. Though MDR/RR-TB is curable using second-line drugs, treatment is complex, long, and generally toxic. In 2023, the treatment success rate for drug-resistant TB was 68% globally^1^. Patient-centered care and support, together with regular patient monitoring to assess regimen effectiveness, is recommended by the World Health Organization (WHO) as a means to facilitate good adherence and ensure improved outcomes^2^.

The consolidated WHO drug-resistant TB guidelines released in 2025 include the option of shorter and longer regimens for treating MDR/RR-TB based on drug resistance profile and other patient characteristics, including age, extent and site of TB disease, and pregnancy status^2^. The 6-month BPaLM regimen (bedaquiline, pretomanid, linezolid, and moxifloxacin) showed an 88% favorable outcome rate in a Phase 3 trial, and in the WHO guidelines is preferred in programmatic conditions over the 9-month regimen^3^. BPaLM is recommended for adults and adolescents aged 14 years or older without fluoroquinolone (FQN) resistance and without extensive TB disease or severe extrapulmonary TB (cases involving the CNS, osteoarticular TB, or disseminated TB). The 6-month BDLLfxC regimen (bedaquiline, delamanid, linezolid, levofloxacin, and clofazimine) is recommended for use in children, adolescents, and pregnant women who cannot benefit from the BPaLM regimen^4,5^. Three additional 9-month all-oral regimens are preferred over longer (18-month) regimens in patients not eligible for the new shorter 6-month regimens^2,6^. On the other hand, individualized longer regimens are recommended for patients with MDR/RR-TB with extensive TB disease, severe forms of extrapulmonary TB, drug intolerance, and for those not eligible or who have not responded well to the previous shorter regimens^2,7^.

In this context, contemporary practice guidelines are beginning to apply stratified medicine principles to TB care such that longer treatments are recommended for the severest forms of disease. Analyses of large databases that encompass the heterogeneity of clinical disease characteristics and treatment composition are needed to adequately inform patient care. Modeling pooled individual participant data from different studies can help to identify clinical and treatment factors associated with unfavorable TB outcomes and permits stratification of patients into different risk phenotypes.

In this pooled analysis of individual participant datasets of patients managed for MDR/RR-TB across a broad geographic distribution, we aimed to identify patient phenotypes at risk of death or treatment failure, and to validate these phenotypes using contemporary trials involving patients treated with an all-oral 6-month regimen composed of bedaquiline, pretomanid, and linezolid (BPaL). A first algorithm was developed to stratify MDR/RR-TB patients according to their risk of unfavorable TB outcome based on pre-treatment clinical characteristics. Additional algorithms were developed to reassess this risk during treatment, considering on-treatment features and culture conversion.

## Results

### Study participants

**Fig. 1:**
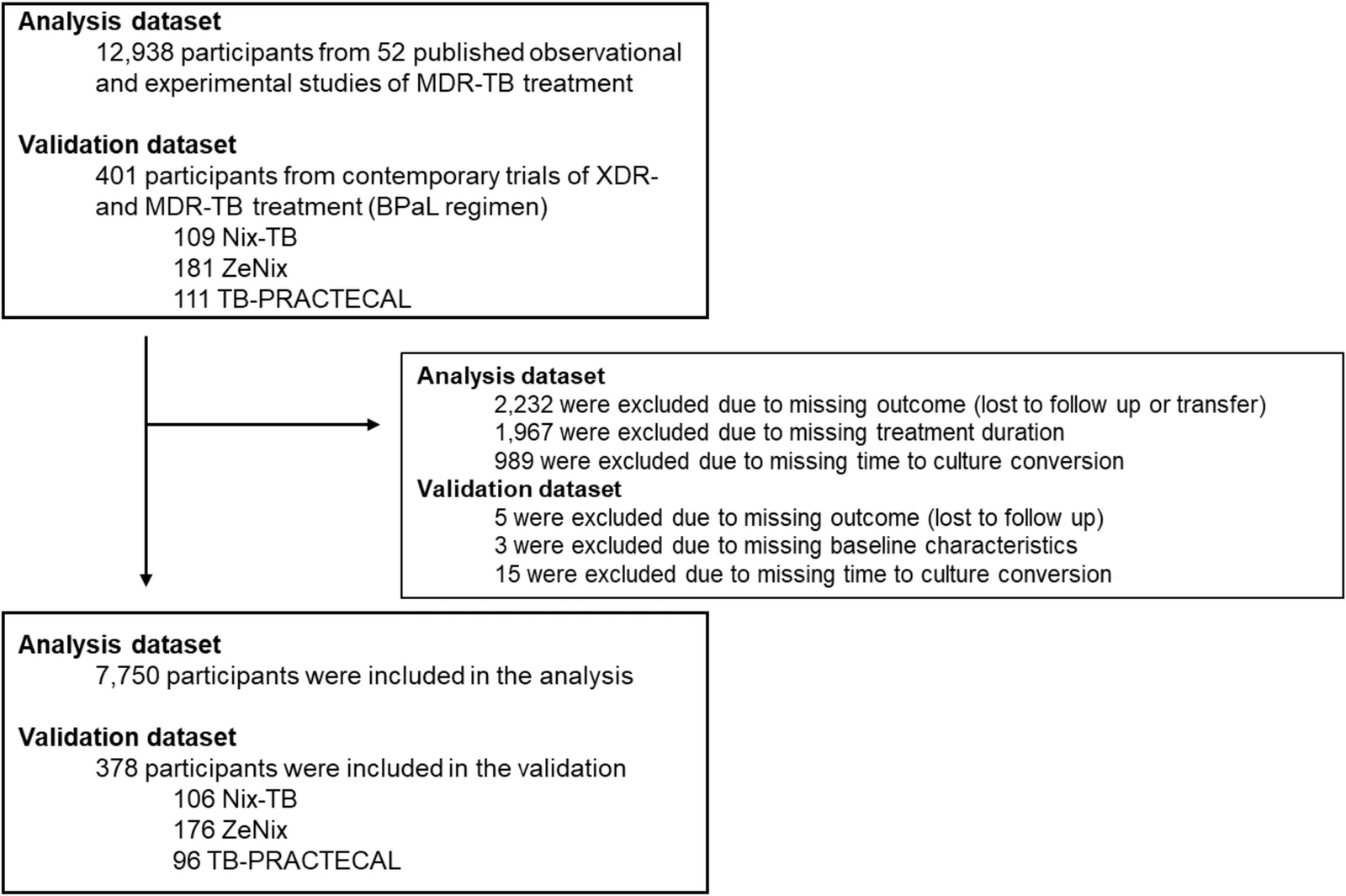
Analysis and validation populations. Data management and inclusion criteria.

The final analysis dataset included 7750 MDR/RR-TB patients, including 5869 patients with treatment success (76%), 628 patients with treatment failure (8%), and 1253 patients who died during the study (16%). The external validation dataset (Nix-TB, ZeNix, and TB-PRACTECAL trials)^8–10^, included 378 patients treated with the 6-month BPaL regimen, with 90% of these patients experiencing treatment success. Figure 1 shows the inclusion criteria.

Participant characteristics are shown for the study population, included in the analysis dataset, and stratified by outcome in Table 1. Overall, the mean age was 37.6 years, mean body mass index (BMI) was 20.23 kg/m^2^, 59.6% were male, and 34.4% were living with HIV, 87% of whom were receiving antiretroviral treatment (ART). Baseline characteristics of participants did not differ across the analysis and validation datasets; participants in the validation dataset had a mean age of 36 years, a mean BMI of 21 kg/m^2^, 61% were male, and 32% HIV positive. Twenty-seven percent of patients in the analysis dataset were resistant to second-line injectables (SLI), and 24.5% resistant to FQNs. In addition, 77% percent of patients in the analysis dataset were ever treated for MDR/RR-TB with a drug for which they had confirmed resistance. The distribution of confirmed resistance to each individual drug ever used in treatment is shown in Supplementary Fig. 1. There was a wide range of drugs used to treat MDR/RR-TB in the analysis dataset, with total treatment duration ranging from 0.1 to 112 months, and a mean value of 19.8 months. Delamanid was used in only 1% of patients, while bedaquiline was used in 21.8%, and linezolid in 19.5% of patients, with the use of the latter two being moderately correlated (55%) (Supplementary Fig. 2).

**Table 1:** Baseline clinical and on-treatment characteristics for the population included in the analysis dataset are shown by treatment outcome (treatment success, treatment failure, death)

	Analysis dataset (*N* = 7750)	TB Outcome		
		Treatment success (*N* = 5869)	Treatment failure (*N* = 628)	Death (*N* = 1253)
**Clinical features**				
Income*				
Low, *n* (%)	1220 (15.7%)	1007 (17.2%)	70 (11.1%)	143 (11.4%)
Middle, *n* (%)	4740 (61.2%)	3298 (56.2%)	430 (68.5%)	1012 (80.8%)
High, *n* (%)	1790 (23.1%)	1564 (26.6%)	128 (20.4%)	98 (7.8%)
**Age**				
N-Miss	1	1	0	0
Mean (SD)	37.63 (12.77)	37.27 (12.79)	38.12 (12.23)	39.05 (12.85)
Median	36	36	37	37
IQR	28–46	2–46	29.75–45	30–47
Range	0–94	0–94	3–82	8–84
**Sex**				
N-Miss	1	1	0	0
Female, *n* (%)	3133 (40.4%)	2354 (40.1%)	243 (38.7%)	536 (42.8%)
**Weight kg**				
N-Miss	3682	2695	238	749
Mean (SD)	55.84 (13.01)	56.98 (12.91)	55.39 (12.58)	49.05 (11.86)
Median	55	56	54.15	48
IQR	47–63	48.3–64.2	47–62	41–56
Range	1.52–164	1.52–164	29–123	23–94
**BMI kg/m**^2^				
N-Miss	4052	2934	258	860
Mean (SD)	20.2 (3.9)	20.48 (3.86)	19.79 (4.12)	18.45 (3.86)
Median	19.8	20.1	19.35	18
IQR	17.6–22.3	18–22.6	17.2–21.9	15.6–20.8
Range	10–55.1	10–55.1	10.9–50.5	10.2–34.3
**Smoking**				
N-Miss	4919	3529	393	997
Yes, *n* (%)	1095 (38.7%)	861 (36.8%)	131 (55.7%)	103 (40.2%)
**Alcohol**				
N-Miss	4424	3111	368	945
Yes, *n* (%)	917 (27.6%)	718 (26.0%)	96 (36.9%)	103 (33.4%)
**HIV status**				
N-Miss	68	53	4	11
Positive, *n* (%)	2641 (34.4%)	1735 (29.8%)	204 (32.7%)	702 (56.5%)
**ART**				
N-Miss	192	96	15	81
Yes, *n*(%)	2314 (30.6%)	1584 (27.4%)	169 (27.6%)	561(47.9%)
**Diabetes mellitus**				
N-Miss	3673	2692	241	740
Yes, *n* (%)	512 (12.6%)	423 (13.3%)	47 (12.1%)	42 (8.2%)
**Past treatment**				
N-Miss	328	241	26	61
No	2586 (34.8%)	2093 (37.2%)	145 (24.1%)	348 (29.2%)
First-line drugs	3409 (45.9%)	2613 (46.4%)	239 (39.7%)	557 (46.7%)
Second-line drugs	1427 (19.2%)	922 (16.4%)	218 (36.2%)	287 (24.1%)
**Site of disease**				
N-Miss	66	47	9	10
Pulmonary	7400 (96.3%)	5604 (96.3%)	607 (98.1%)	1189 (95.7%)
Extrapulmonary	284 (3.7%)	218 (3.7%)	12 (1.9%)	54 (4.3%)
**Cavity present**				
N-Miss	3399	2451	213	735
Yes, *n* (%)	2659 (61.1%)	2002 (58.6%)	307 (74.0%)	350 (67.6%)
**Bilateral disease**				
N-Miss	4294	3069	343	882
Yes, *n* (%)	2396 (69.3%)	1856 (66.3%)	233 (81.8%)	307 (82.7%)
**AFB smear**				
N-Miss	523	411	25	87
Positive, *n* (%)	5205 (72.0%)	3885 (71.2%)	490 (81.3%)	830 (71.2%)
**DSTE**				
N-Miss	3310	2240	256	814
Resistant, *n* (%)	3107 (70.0%)	2483 (68.4%)	293 (78.8%)	331 (75.4%)
**DSTEtoPto**				
N-Miss	4182	2910	318	954
Resistant, *n* (%)	1126 (31.6%)	924 (31.2%)	122 (39.4%)	80 (26.8%)
**DSTPZA**				
N-Miss	4319	2970	380	969
Resistant, *n* (%)	1887 (55.0%)	1531 (52.8%)	176 (71.0%)	180 (63.4%)
**DSTS**				
N-Miss	3109	2105	246	758
Resistant, *n* (%)	3452 (74.4%)	2749 (73.0%)	305 (79.8%)	398 (80.4%)
**DSTFQN**				
N-Miss	245	184	25	36
Resistant, *n* (%)	1841 (24.5%)	1128 (19.8%)	286 (47.4%)	427 (35.1%)
**DSTSLI**				
N-Miss	232	179	24	29
Resistant, *n* (%)	2057 (27.4%)	1313 (23.1%)	279 (46.2%)	465 (38.0%)
**DSTPAS**				
N-Miss	4735	3382	351	1002
Resistant, *n* (%)	542 (18.0%)	430 (17.3%)	80 (28.9%)	32 (12.7%)
**On-treatment features** **Six-month culture conversion**				
Yes, *n* (%)	6337 (81.8%)	5511 (93.9%)	239 (38.1%)	587 (46.8%)
**Treatment duration (months)**				
Mean (SD)	19.76 (7.57)	21.84 (4.31)	20.13 (11.36)	9.84 (9.15)
Median	20.6	21.5	21.2	7.7
IQR	18–24	19.1–24	11.5–26.15	2.6–17.4
Range	0.1–112	6.1–68.9	0.1–61.7	0.1–112
**Drugs ever used**				
E, *n* (%)	3563 (46.0%)	2622 (44.7%)	310 (49.4%)	631 (50.4%)
PZA, *n* (%)	6312 (81.4%)	4672 (79.6%)	520 (82.8%)	1120 (89.4%)
S, *n* (%)	657 (8.5%)	546 (9.3%)	42 (6.7%)	69 (5.5%)
INJ, *n* (%)	6664 (86.0%)	5060 (86.2%)	539 (85.8%)	1065 (85.0%)
FQN, *n* (%)	7200 (92.9%)	5593 (95.3%)	544 (86.6%)	1063 (84.8%)
EtoPto, *n* (%)	6136 (79.2%)	4609 (78.5%)	499 (79.5%)	1028 (82.0%)
CsTrd, *n* (%)	6586 (85.0%)	5077 (86.5%)	505 (80.4%)	1004 (80.1%)
MpmIpm, *n* (%)	214 (2.8%)	183 (3.1%)	11 (1.8%)	20 (1.6%)
PAS, *n* (%)	3269 (42.2%)	2343 (39.9%)	368 (58.6%)	558 (44.5%)
Lzd, *n* (%)	1515 (19.5%)	1235 (21.0%)	112 (17.8%)	168 (13.4%)
Cfz, *n* (%)	997 (12.9%)	763 (13.0%)	84 (13.4%)	150 (12.0%)
Bdq, *n* (%)	1692 (21.8%)	1329 (22.6%)	144 (22.9%)	219 (17.5%)
Dlm, *n* (%)	66 (0.9%)	61 (1.0%)	2 (0.3%)	3 (0.2%)

*N-Miss* number of participants with missing values, *IQR* interquartile range, *ART* people living with HIV on antiretroviral treatment, *AFB* acid-fast bacilli, *DST* drug susceptibility test, *E* ethambutol, *PZA* pyrazinamide, *S* streptomycin, *EtoPto* ethionamide or prothionamide, *CsTrd* cycloserine or terizidone, *Cfz* clofazimine, *Lzd* linezolid, *Bdq* bedaquiline, *Dlm* delaminid, *MpmIpm* meropenem or imipenem, *FQN* fluoroquinolones, *INJ* injectable drugs, *SLI* second-line injectables.

*Income calculated based on the WHO country Income.

Notably, of the 16% of patients who died, 56.5% were HIV positive. There were also notable differences between treatment duration across outcomes. In addition, 6-month culture conversion rates were higher in patients who experienced treatment success than those patients who experienced treatment failure or death (*P* < 0.001) (Table 1).

### Baseline clinical predictors of death and failure

Multivariable logistic regression analysis of baseline risk factors showed that both treatment failure and death were associated with BMI (aORs are reported per unit increase in BMI: Death aOR, 0.87; 95% CI, 0.82–0.92. Failure aOR, 0.94; 95% CI, 0.91–0.98), previous TB treatment with second-line drugs (Death aOR, 1.45; 95% CI, 1.01–2.07. Failure aOR, 1.61; 95%CI, 1.21–2.15), cavitary disease (Death aOR, 1.46; 95% CI, 1.10–1.94. Failure aOR, 1.57; 95% CI, 1.15–2.13) and pyrazinamide resistance (Death aOR, 1.68; 95% CI, 1.30–2.17. Failure aOR 1.63; 95% CI, 1.22–2.17). Death was also associated with HIV infection (aOR, 2.24; 95% CI, 1.84–2.73), older age (aOR per year increase, 1.03; 95% CI, 1.02–1.03), males (Females aOR, 0.69; 95% CI, 0.52–0.92), and extrapulmonary disease (aOR, 1.78; 95% CI, 1.24–2.56), while treatment failure was associated with positive sputum acid-fast bacillus (AFB) smear (aOR, 1.74; 95% CI, 1.35–2.24), smoking (aOR, 1.79; 95% CI, 1.21–2.65), and FQN resistance (aOR, 2.11; 95% CI, 1.66–2.68), or SLI (aOR, 1.30; 95% CI, 1.03–1.63) (Supplementary Fig. 3).

### Risk stratification analysis

**Fig. 2:**
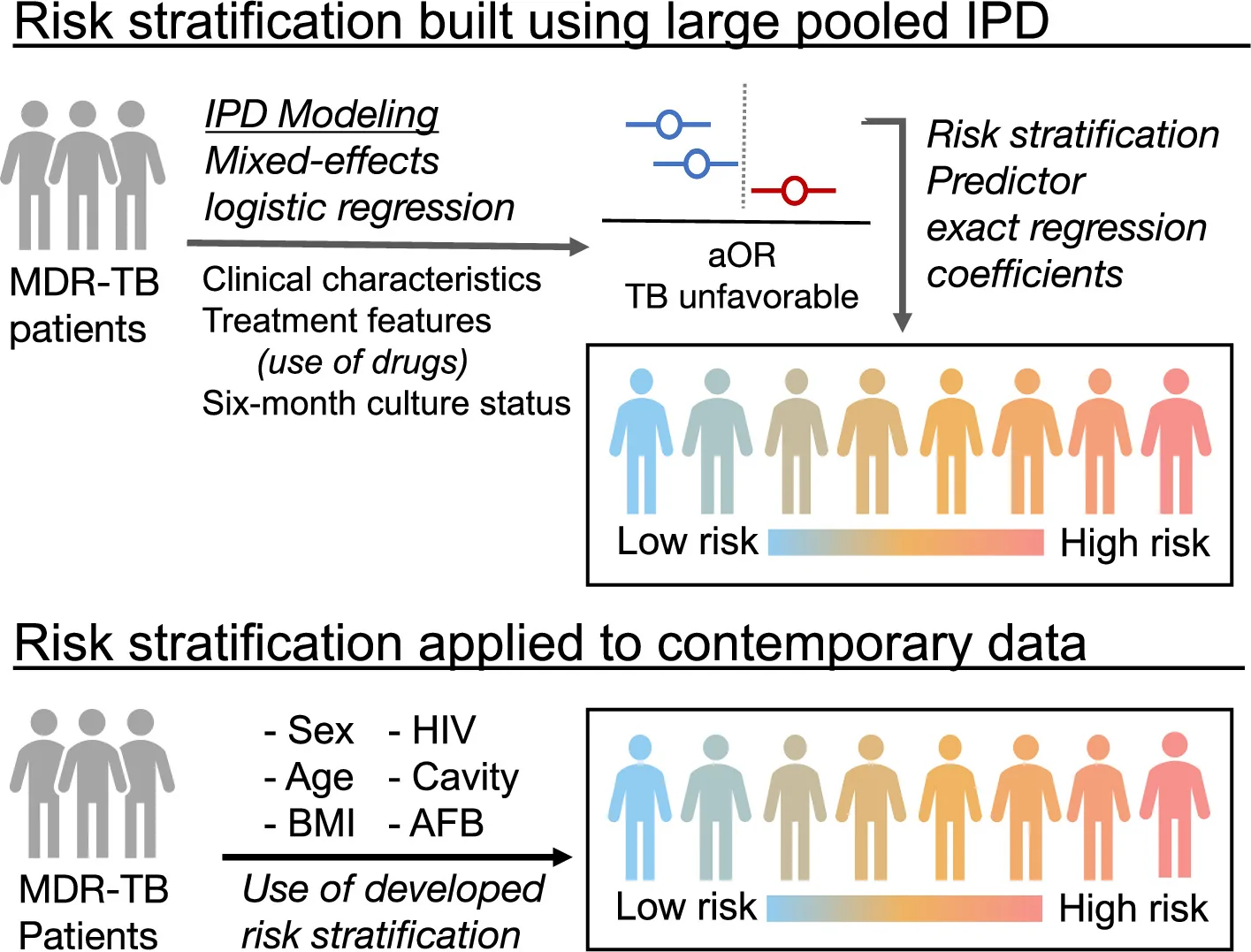
Diagram representing the development and application of the risk stratification algorithm. aOR adjusted odds ratio.

Figure 2 provides a high-level overview of the approach followed in the current analysis for constructing the risk stratification algorithm using the analysis dataset and its application in stratifying contemporary TB patients into different phenotypes.

**Fig. 3:**
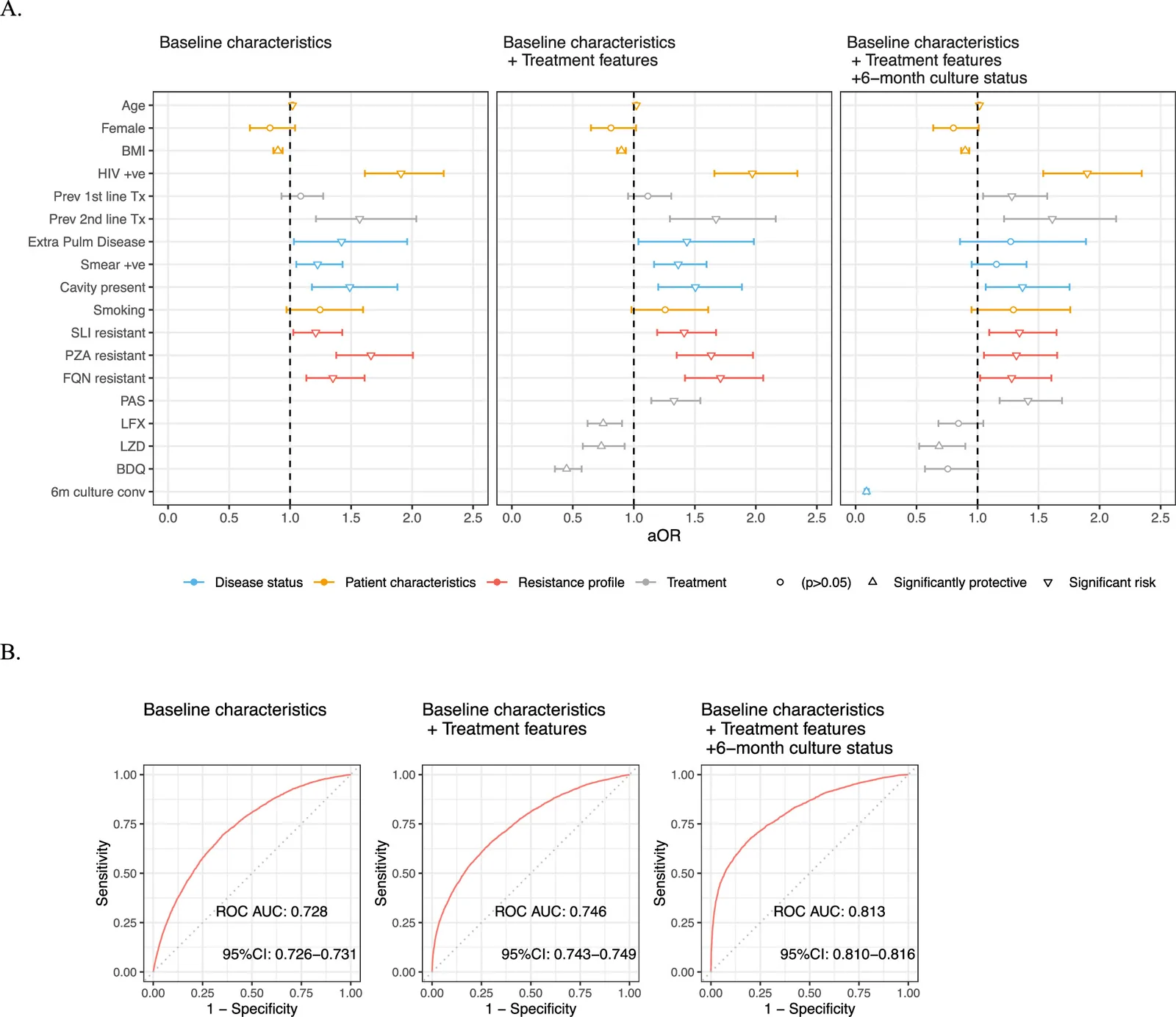
Models to predict the probability of unfavorable TB outcome and their evaluation. **A** Multivariable analysis for unfavorable outcomes of the clinical baseline and on-treatment predictors for Model A (Baseline characteristics), Model B (Baseline characteristics + Treatment features), and Model C (Baseline characteristics + Treatment features +6-month culture status) built using mixed-effects logistic regression based on the analysis dataset (*n* = 7750 individuals for Models A and B; *n* = 7109 individuals for Model C, excluding patients who died or had treatment failure before 6 months of treatment). Adjusted odds ratios (aOR) with 95% CIs are reported, with the center defined as the aOR and error bars representing the 95% CIs. Age and BMI were modeled as continuous variables; their aORs represent the change in odds of the outcome per one-year increase in age and per one-unit increase in BMI (kg/m²), respectively; all other variables were modeled as categorical. Statistical significance was assessed using two-sided Wald tests derived from the mixed-effects logistic regression models; estimates were pooled across imputed datasets following Rubin´s rules using the mice package; no adjustment for multiple comparisons was applied. **B** ROC curve plots for the three models developed using all data (Model A and B), and utilizing data that excluded patients who died or had treatment failure before 6 months of treatment (Model C). HIV +ve HIV positive, Prev 1st line Tx patient received past TB treatment with first-line drugs, Prev 2nd line Tx patient received past TB treatment with second-line drugs, Extra Pulm Disease extrapulmonary disease, Smear +ve smear positive, SLI second-line injectables, PZA pyrazinamide, FQN fluoroquinolones, LZD linezolid, BDQ bedaquiline, LFX levofloxacin, PAS para-aminosalicylic acid, 6 m culture conv 6-month culture conversion.

Following model comparison, the mixed-effects logistic regression model was selected for risk stratification, as it consistently outperformed mixed-effects gradient boosting (GPBoost) models in cross-validation and independent testing (Supplementary Table 1 and Supplementary Fig. 4). Baseline clinical features included in the risk stratification models, and their effect on TB unfavorable outcome are displayed in Fig. 3A. The inclusion of individual drugs improved model discrimination with an increase in area under the receiver operating characteristic curve (ROC AUC) from 0.728 (95% CI, 0.726–0.731) (Model A, built using baseline characteristics) to 0.746 (95% CI, 0.743–0.749) (Model B, built using baseline characteristics and treatment features) (Fig. 3B). The use of linezolid (aOR, 0.73; 95% CI, 0.58–0.90), bedaquiline (aOR, 0.45; 95% CI, 0.35–0.57), and levofloxacin (aOR, 0.75; 95% CI, 0.62–0.99), were significantly associated with lower odds of unfavorable TB outcome, whereas the use of PAS (aOR, 1.33; 95% CI, 1.14–1.54) was associated with higher odds of unfavorable outcome (Fig. 3A).

HIV seropositivity was the baseline risk factor most strongly associated with odds of unfavorable outcomes, with an adjusted OR of 1.91 (95% CI, 1.61–2.26). Six-month culture conversion was the strongest predictor associated with reduced odds of unfavorable outcomes (aOR, 0.097; 95% CI, 0.07–0.11), while improving model discrimination with an increase in ROC AUC from 0.733 (95% CI, 0.730–0.737) (Model B, built using baseline characteristics and treatment features and applied in patients with treatment duration longer than 6 months) to 0.813 (95% CI, 0.810–0.816) (Model C, built using baseline characteristics, treatment features and 6-month culture status) (Fig. 3B).

**Fig. 4:**
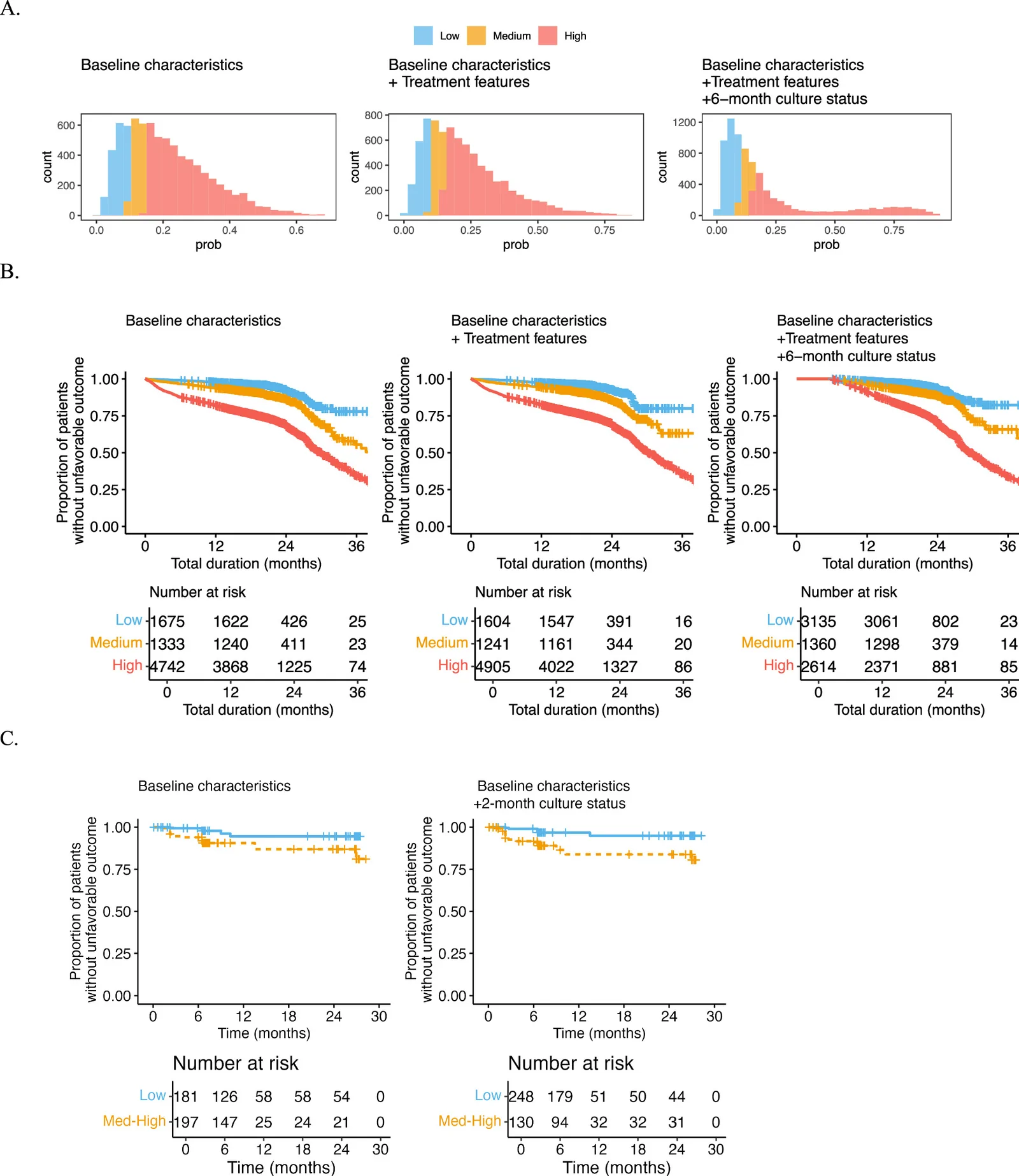
Risk stratification analysis. **A** Predicted probability of unfavorable outcomes distribution in the analysis dataset for the different models. **B** Probability of unfavorable outcome over time since start of treatment based on the risk stratification, in the analysis dataset, using models A, B, and C. Example showing data imputed number 1. Model C representation excluded patients who died or had treatment failure before 6 months of treatment. **C** Probability of unfavorable outcome over time since start of treatment based on the risk stratification, in the validation dataset with participants receiving BPaL regimen as part of their treatment schedule, using models A and C.

The predicted probabilities of unfavorable TB outcomes obtained with Models A, B and C are displayed in Fig. 4A.The proportion of patients with treatment success (i.e., no unfavorable outcome) over time since treatment initiation was different (*P* < 0.001, log-rank test) between low (predicted probabilities of an unfavorable TB outcome less than 10%), medium (predicted probabilities of an unfavorable TB outcome between 10 and 15%), and high-risk phenotypes (predicted probabilities of an unfavorable TB outcome greater than 15%) (Fig. 4B).

The risk stratification algorithm using pre-treatment clinical characteristics (Model A) and on-treatment marker culture conversion (Model C) was externally validated using contemporary data from patients who received the BPaL regimen. The proportion of patients with no unfavorable outcome over time since treatment initiation differed between low-risk and medium and high-risk phenotypes (Model A, *P* = 0.0059; Model C, *P* = 0.0012; log-rank test) (Fig. 4C). At the end of treatment, the proportion of treatment success was 97% with Model A and 97% with Model C in the low-risk phenotype, and 91% with Model A and 88% with Model C in the medium and high-risk phenotypes (Fig. 4C).

Baseline characteristics were strong predictors of low-risk phenotypes, as the distribution of patients in the low-risk category, with 91% of patients initially categorized as low risk remaining so at 6 months, and only 8% moving to high risk at 6 months. Among medium-risk patients, 13% moved to high risk and 71% to low risk at 6 months. For high-risk patients, 20% switched to low risk and 27% to medium risk at 6 months (Supplementary Fig. 5A). Similarly, using a validation dataset with contemporary patients and treatments, 52% of medium-high-risk patients were re-categorized as low risk after 2 months of treatment, while 81% of those who were initially low risk remained low risk (Supplementary Fig. 5B).

When looking at the risk groups established at the start of treatment in the analysis dataset, bedaquiline and linezolid were most widely used in the high-risk groups (30.9%% and 25.5%, respectively) compared to the low-risk groups (10.7% and 12.7%, respectively) (Supplementary Table 2). These are unadjusted frequencies showing that LZQ and BDQ were more frequently used in patients categorized as high risk at baseline. After adjustment for baseline characteristics, both drugs were independently associated with a lower risk of unfavorable outcomes (Fig. 3A).

## Discussion

In this study, we used pooled individual data to develop a risk stratification algorithm that stratified MDR/RR-TB patients into low-, medium-, and high- risk groups at the start of treatment based on baseline clinical characteristics, and reassessed their risk categorization after the initiation of treatment using on-treatment features. Our definitions of risk groups were validated in an independent contemporary, pooled 3-trial dataset of patients receiving a 6-month BPaL regimen for MDR/RR-TB. With these risk stratification algorithms, we identified patients at low and high risk for unfavorable TB outcomes.

These findings are consistent with and build upon previous studies^11–18^. For example, HIV co-infection, lower baseline BMI, positive baseline sputum smear grade, and presence of cavities have been previously identified as predictors of delayed culture conversion among MDR/RR-TB patients^13,14^, which in turn was associated with TB treatment outcomes. Of note, MDR/RR-TB treatment does not usually differ for people living with HIV or patients with low BMI, but in this study HIV was the most important baseline clinical predictor of TB unfavorable outcome (aOR 1.91; 95%CI 1.61–2.26; *P* < 0.001, Fig. 3A) and BMI had a strong association with TB treatment success (aOR per unit increase 0.90; 95%CI 0.86–0.94; *P* < 0.001, Fig. 3A), with both variables having a significant impact on participants’ risk assignment. While HIV positivity was linked to unfavorable outcomes, people living with HIV not on ART showed a higher risk (aOR 2.4, 95% CI 1.7–3.4) compared to those on ART (aOR 1.8, 95% CI 1.5–2.2), similar to what has been reported in other studies^15^. However, even among those receiving ART, a significant risk of TB unfavorable outcomes persisted. Resistance to FQN was also considered in the risk algorithm, given its established importance in current MDR/RR-TB guidelines and treatment response^7,19–21^, and its association with failure and TB unfavorable outcome in the overall analysis.

The development of Model C allowed for a reassessment of patient risk after the initiation of treatment based on culture conversion data. Six-month culture conversion was strongly associated with treatment success in patients treated for MDR/RR-TB^11,22^. Our analysis showed that patients who were identified as at low risk of unfavorable TB outcome were more likely to remain at low-risk status at a 6-month risk re-evaluation. However, those patients identified as medium risk and high risk could benefit from this risk re-evaluation depending on their culture conversion status. The practical value of Model C lies in its ability to capture real-world heterogeneity in TB trial endpoints and supports the use of culture positivity to guide treatment intervention.

Tuberculosis management is complex due to the diverse disease burden and severity of the disease, with a wide range of patient phenotypes^23,24^. While standard treatment is overall effective, disease management based on a one-size-fits-all approach can result in the undertreatment of patients with extensive disease and lead to unnecessarily long treatment with potential toxicities for many patients with less severe disease. This scenario is even more complex in the case of MDR/RR-TB patients, whose treatment duration is longer, with high variability in resistance profiles to different TB drugs, and poorer treatment prognosis^25^. Fortunately, MDR/RR-TB disease management has changed from longer to shorter treatments in recent years^7,19^ In addition, new diagnostic tools that simplify or enhance drug resistance testing can provide targeted treatment to these patients with drug resistance^26–28^.

The integration of individual patient data represents an opportunity to provide a more individualized treatment and target patients early in treatment who could benefit from early treatment interventions to maximize their cure rates. For example, a recent patient-level pooled analysis of Phase 3 clinical trials that evaluated shorter treatments for drug-susceptible TB revealed that patients with non-cavitary disease and low sputum burden could be successfully treated in 4 months rather than 6 months^29^. Based on those results, the authors developed a risk stratification algorithm and clinical simulation tool using six markers of risk routinely collected in clinical trials, which were also included in our risk model, (HIV status, baseline smear grade, sex, baseline cavitary disease, baseline BMI, and 2-month culture status) to provide more individualized predictions of optimal treatment regimens to achieve high cure rates in TB patients^29,30^. Following this example, we can provide better suggestions for individualized treatment in MDR/RR-TB patients based on quantifiable data and use our analysis to target MDR/RR-TB patient phenotypes. Of note, the study of patient phenotypes is not novel, and similar approaches, integrating data from several studies to identify different patient phenotypes and matching them to the most appropriate treatment, have been applied in other therapeutic areas, such as HIV prevention^31^.

Our study has limitations. First, the limited availability of post-treatment completion data in the analysis dataset reduced our ability to capture recurrence information, a crucial endpoint when considering treatment interventions such as shortened treatment durations. Consequently, our analysis was confined to data collected during the treatment period. Nevertheless, we successfully validated the risk algorithm using data from contemporary trials that included post-treatment follow-up and relapse data. Second, a significant number of individuals were lost to follow-up during treatment and thus excluded from the main analysis, restricting our study to only those with known outcomes at the end of treatment; this may also have introduced bias, as loss to follow-up might be more likely to mask treatment failure and death. Third, our study included data published through 2018, with the latest treatment initiation date in 2016, and did not include data on the newer regimens recently recommended in the 2025 guideline^2^. Nonetheless, given that the risk algorithm uses features routinely collected in TB clinical trials, we were able to validate the analyses with recent data from patients treated with newer, all-oral, shorter 6-month treatment regimens for MDR/RR-TB and pre-extensively drug-resistant (pre-XDR-TB)^8–10^. In addition, in the external validation datasets, 2-month culture conversion was used as a proxy for 6-month conversion due to data availability, which may have introduced some attenuation of predictive performance.

Our study also has several strengths. We integrated large quantifiable data from 52 MDR/RR-TB datasets with patients managed for MDR/RR-TB across a broad geographic distribution. While previous research has extensively examined this dataset^21^, it has not been previously employed to identify patient phenotypes and stratify them based on their predicted risk of unfavorable TB outcomes. To the best of our knowledge, this is the first risk stratification algorithm for MDR/RR-TB patients utilizing a wide variety of individual information, including demographic and disease characteristics, drug susceptibility information, treatment composition, and outcomes. In addition, our algorithm was applied to an external contemporary dataset, demonstrating its potential to effectively assess individual risk of unfavorable TB outcomes and enabling the identification of patient risk phenotypes. This personalized approach allows for the tailoring of treatment strategies to better suit the needs of each patient. Risk stratification algorithms hold promise for guiding the design of future clinical trials in MDR/RR-TB, facilitating the testing of personalized treatment options across different patient populations based on their pre-treatment clinical and demographic characteristics. Moreover, such algorithms offer the opportunity for a reassessment of risk during treatment, incorporating on-treatment factors such as culture conversion, thereby enhancing the precision of treatment interventions. The work presented here forms the basis for a new Phase 2C/3 clinical trial (PRISM-TB, ClinicalTrials.gov NCT06441006), by the U.S. Department of State-funded SMART4TB consortium, aimed at prospectively validating a prespecified treatment duration stratification algorithm for the treatment of FQN-susceptible MDR/RR-TB.

In conclusion, our validated analyses of individual participant data of a large dataset provide a risk stratification algorithm for patients with MDR/RR-TB that stratifies patients at the start of treatment and re-evaluates their risk after 6 months of treatment. Our results indicate that stratified medicine approaches where interventions can be applied early in treatment in relation to low, medium, and high-risk phenotypes can be used to enhance cure for MDR/RR-TB patients.

## Methods

### Ethics statement

This research complies with all relevant ethical regulations. Assembly and use of this individual patient data was approved by the Research Institute of the McGill University Health Centre (BMB-07-021t), as well as by participating sites, as necessary. Informed consent was obtained from all participants at the time of original data collection by the contributing studies. Data from the three Phase 3 trials used for the external validation (Nix-TB, NCT02333799; ZeNix, NCT03086486; and TB-PRACTECAL, NCT02589782) were collected under protocols reviewed and approved by ethics committees and regulatory committees described in their original publications^8–10^ with written informed consent obtained from all participants.

### Study rationale

The primary objective of this study was to stratify MDR/RR-TB patients into risk phenotypes based on model-estimated probabilities of unfavorable outcomes. For this purpose, large pooled individual patient data was modeled to identify clinical characteristics that could be used as predictors of treatment unfavorable outcome, building a risk stratification algorithm (Fig. 2). In addition, treatment characteristics, including individual drugs given ever along the treatment period, were accounted for risk re-evaluation after treatment initiation and, particularly, 6-month culture status, was also evaluated as a predictor of treatment outcome for risk re-evaluation 6 months after the initiation of treatment. This risk stratification algorithm was then validated against contemporary datasets, which included highly effective treatments, to ensure its applicability in current clinical practices (Fig. 2).

### Data source

Individual-level data from 12,938 patients with pulmonary TB and confirmed rifampin resistance from 52 observational and experimental studies of MDR/RR-TB treatment performed across 38 countries/regions and collected between 2009 and 2018^21^, were considered for the analysis. The dataset was described in detail elsewhere^21^. In brief, the database included (1) studies of treatment of MDR/RR-TB, published between Jan 1, 2009, and April 15, 2016, identified from a systematic review^32^, (2) data from investigators who contributed to an individual patient data meta-analysis conducted in 2010^33^, (3) and new data from a public call performed by WHO in 2018^34^. For eligibility, studies had to report original results, with outcomes at the end of treatment in cohorts of at least 25 adults (aged >18 years). The dataset included patients who initiated treatment between 1993 and 2016, and provided information on pre-treatment clinical and demographic characteristics, treatment features (individual drugs given ever along the treatment period and total treatment duration), 6-month culture conversion, and treatment outcomes. Patients who were lost to follow-up were excluded from the main analysis (supported by the sensitivity analysis reported in Supplementary Fig. 6), as well as those with missing data on-treatment duration and time to culture conversion. TB recurrence data were not considered because there was not enough data (<1% patients with such information). The dataset was split into training (80%) and testing (20%) sets, stratified by study. Baseline characteristics of interest that were missing (age, sex, weight, BMI, smoking, alcohol use, HIV status, diabetes mellitus, past treatment, site of the disease, cavity, bilateral disease, AFB and TB drug susceptibility test) were assumed to be missing at random and were imputed from the other characteristics and outcome data, as well as study characteristics (year, country-level income) using multivariate imputation via chained equations (20 imputed datasets), using the R package mice^35^. The imputation approach was developed using the training dataset and applied to the testing dataset to avoid data leakage. Each analysis was performed separately on the imputed datasets, and parameter estimates and standard errors were combined using Rubin’s rules to properly account for the uncertainty due to imputation.

Data from three Phase 3 trials, Nix-TB (NCT02333799) performed between 2015 and 2019, ZeNix (NCT03086486) performed between 2017 and 2021, and TB-PRACTECAL (NCT02589782) performed between 2017 and 2022, that tested all-oral shorter treatment regimens of 6-months BPaL regimen in patients with XDR- and MDR-TB, were obtained directly from the trial sponsors and were used to externally validate the model^8–10^, after excluding those who were lost to follow-up.

### Efficacy outcomes and analysis approach

The primary efficacy endpoint was unfavorable TB outcome (defined as treatment failure or death), compared to treatment success (defined as cure or completion). These treatment outcomes (treatment failure, death, and treatment success) were defined as described in the original meta-analysis manuscript, in which individual studies used either the WHO or Laserson and colleagues definitions, depending on their original reporting^21,36,37^ (Supplementary Table 3). Unfavorable TB outcome definition in the validation dataset included relapse observed 12–18 months post-treatment completion in addition to treatment failure or death.

We also developed separate baseline models for treatment success compared to (i) death, or (ii) failure, to understand if there were clinical baseline characteristics that were associated only with death, or only with failure. In the model evaluating treatment success versus death, patients who had experienced treatment failure were excluded. Conversely, in the model evaluating treatment success versus treatment failure, patients who died were excluded.

Three multivariable models were developed: Model A—a baseline model that included pre-treatment clinical and demographic predictors associated with unfavorable TB outcome. Model B—an on-treatment model that included the individual drugs ever used as part of the treatment that were significantly associated with treatment outcome during model building, in addition to pre-treatment clinical predictors; and Model C—an on-treatment model that included six-month culture conversion (defined as two consecutive negative sputum cultures, at least 30 days apart), in addition to pre-treatment clinical characteristics and drugs used. Model C was developed excluding patients who died or had treatment failure before 6 months of treatment. Participants with culture-negative at baseline or who were converted by 6 months were included in the group who converted at 6 months.

### Model development, cross-validation, selection, and evaluation

Each efficacy endpoint (any unfavorable TB outcome, death, or failure) was analyzed to identify predictors of each endpoint. Each model was adjusted for pre-treatment characteristics associated with outcomes and World Bank country income category as fixed effects, as well as including study and center as random intercepts. Multivariable models were built using a stepwise fashion with forward inclusion (*P* < 0.05 to include) then backward deletion (*P *> 0.01 to remove), using as a reference starting point the predictors included in the drug-susceptible pulmonary TB risk stratification model^29,30^, as well as those that showed significance in a first preliminary univariable analysis. Individual drugs used for treatment of MDR/RR-TB were also tested in a stepwise fashion with forward inclusion (*P* < 0.05 to include), then backward deletion (*P* > 0.01 to remove).

Within each imputed training dataset, a fivefold cross-validation was performed. Mixed-effects logistic regression and GPBoost models were trained and evaluated using identical sets of predictors to allow fair comparison. For GPBoost, hyperparameters were tuned using nested cross-validation within the training folds. Model discrimination was assessed using ROC AUC. After cross-validation, both modeling approaches were refit using the full training dataset and evaluated on the independent testing dataset. GPBoost models were implemented using the gpboost package (version 1.5.8) in R. Train/test splitting and cross-validation were performed only for the comparison of predictive performance between the boosted regression tree and logistic regression models. The model with better performance was selected to fit the full analysis dataset as final models.

Model evaluation for the three final models (A, B, C) was performed using all data for Model A and B, and utilizing data excluding patients who died or had treatment failure before 6 months of treatment for Model C. In this line, and only to enhance model discrimination with the incorporation of 6-month culture conversion, Model B underwent re-estimation using data excluding patients who encountered treatment failure or mortality before completing 6 months of treatment, facilitating ROC AUC comparison.

### Risk stratification algorithm

The exact regression coefficients of the baseline and on-treatment predictors of TB-related outcomes were used to derive the probability of unfavorable TB outcome for each individual, in the analysis (using models A, B, and C) and in the validation (using models A and C) datasets. In Model C, 6-month culture conversion was included as an on-treatment predictor. In the external validation datasets, information on 6-month culture conversion was unavailable; instead, 2-month culture conversion was recorded. For validation purposes, we therefore used the coefficient estimated for the 6-month conversion in Model C and applied it to the corresponding 2-month conversion variable, assuming both markers capture a similar underlying treatment-response process. This was supported by re-fitting the model on the validation data and by showing that the coefficient for 2-month conversion was very close to the one using 6-month conversion (Supplementary Table 4).

In addition, the treatment effect was not considered since all patients in the validation database received the same drugs. We explored the distribution of probabilities for the three models in the analysis dataset and assigned definitions of low, medium, and high-risk groups by classifying patients with predicted probabilities of an unfavorable TB outcome of less than 10% as low risk, those between 10 and 15% as medium risk, and those with greater than 15% as high risk. Risk cut-offs were derived from the distribution of predicted probabilities in Model A, which includes only pre-treatment characteristics, allowing risk stratification to be performed before treatment initiation. These thresholds corresponded to the 33rd and 66th percentiles of the Model A probability distribution. In the case of the validation database, the medium and high-risk groups were pooled due to limitations of data available in the individual groups. Using this stratification and examining treatment duration, the proportion of unfavorable treatment outcomes over time since the start of treatment was assessed for the three risk groups.

### Reporting summary

Further information on research design is available in the Nature Portfolio Reporting Summary linked to this article.

## Supplementary information

Supplementary Information

Reporting Summary

Transparent Peer Review file

## Data Availability

The individual patient data used in this study were part of the WHO TB treatment individual patient data (TB-IPD) platform, a global data repository jointly established by the World Health Organization Global TB Programme and University College London (UCL). Access to these data is subject to approval by the Data Access Committee (see https://www.ucl.ac.uk/global-health/research/z-research/tb-ipd-platform for further details). Data from the Phase 3 trials used for external validation (Nix-TB, ZeNix, and TB-PRACTECAL) are available from the respective trial sponsors, subject to their data-sharing policies.

## Code availability

All data processing and analysis were conducted using the R statistical software version 3.6.2. Upon request, the authors will provide the code for the modeling analysis that supports the findings of this study.
