# Supplementary material for: Risk stratification for MDR/RR-TB: a comprehensive integrated patient-level analysis from TB programs and contemporary clinical trials
Source: Nat Commun. 2026 Sep 22;17:11337. doi: 10.1038/s41467-026-77931-0 (PMC13728123; doi:10.1038/s41467-026-77931-0)
Supplement: Supplementary file 1 [file 41467_2026_77931_MOESM1_ESM.pdf]

SUPPLEMENTARY INFORMATION

Supplementary Information to: Risk Stratification for MDR/RR-TB: A Comprehensive Integrated Patient-Level Analysis from TB Programs and Contemporary Clinical Trials

Supplementary Figure 1. Distribution of confirmed resistance against the drugs ever used on treatment in participants treated with those drugs.

Supplementary Figure 2. Drug-use combination correlation.

Supplementary Figure 3. Multivariable aORs for death, treatment failure, and TB unfavorable outcome (death and treatment failure) of the clinical baseline predictors.

Supplementary Figure 4. The boxplot of ROC AUC of the mixed-effect logistic regression model and the mixed-effect gradient boosting model in the testing dataset (n=1629) among all 20 imputations.

Supplementary Figure 5. Comparison of models on risk stratification re-evaluations.

Supplementary Figure 6. Sensitivity analyses evaluating clinical predictors of TB unfavorable outcome with participants with a lost to follow-up outcome considered either to have a successful or an unfavorable TB treatment outcome.

Supplementary Table 1. ROC AUC performance of all models in the validation dataset during 5-fold cross-validation.

Supplementary Table 2. Proportion of drug use by risk strata (using Model A- Baseline characteristics, n=7750).

Supplementary Table 3. Treatment outcomes definitions for MDR/RR-TB patients.

Supplementary Table 4. Six-Months culture conversion and 2-Months culture conversion coefficients comparison.

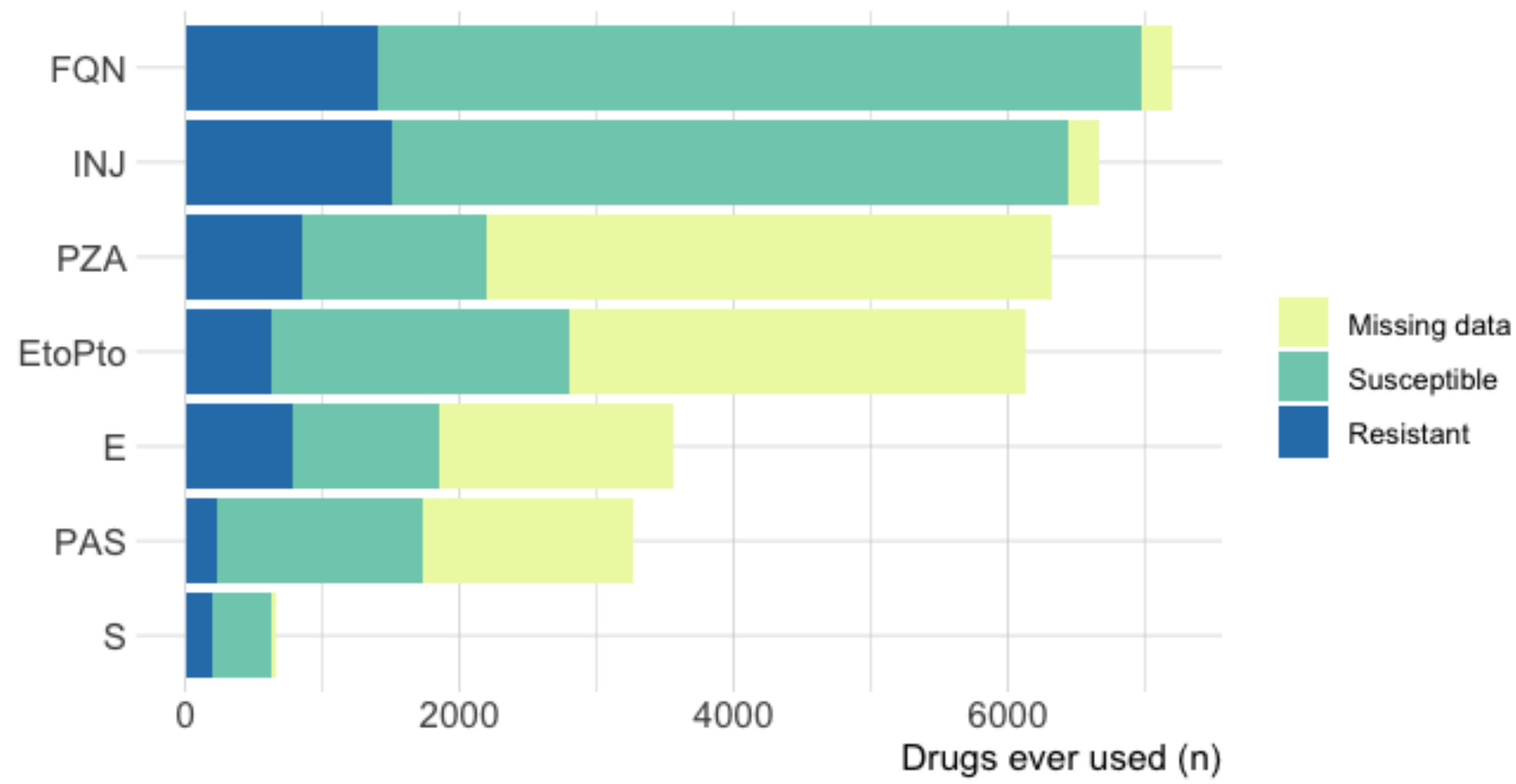

Supplementary Figure 1. Distribution of confirmed resistance against the drugs ever used on treatment in participants treated with those drugs.

E, Ethambutol. PZA, Pyrazinamide. S, Streptomycin. EtoPto, Ethionamide or Prothionamide. FQN, fluoroquinolones. INJ, injectable drugs.

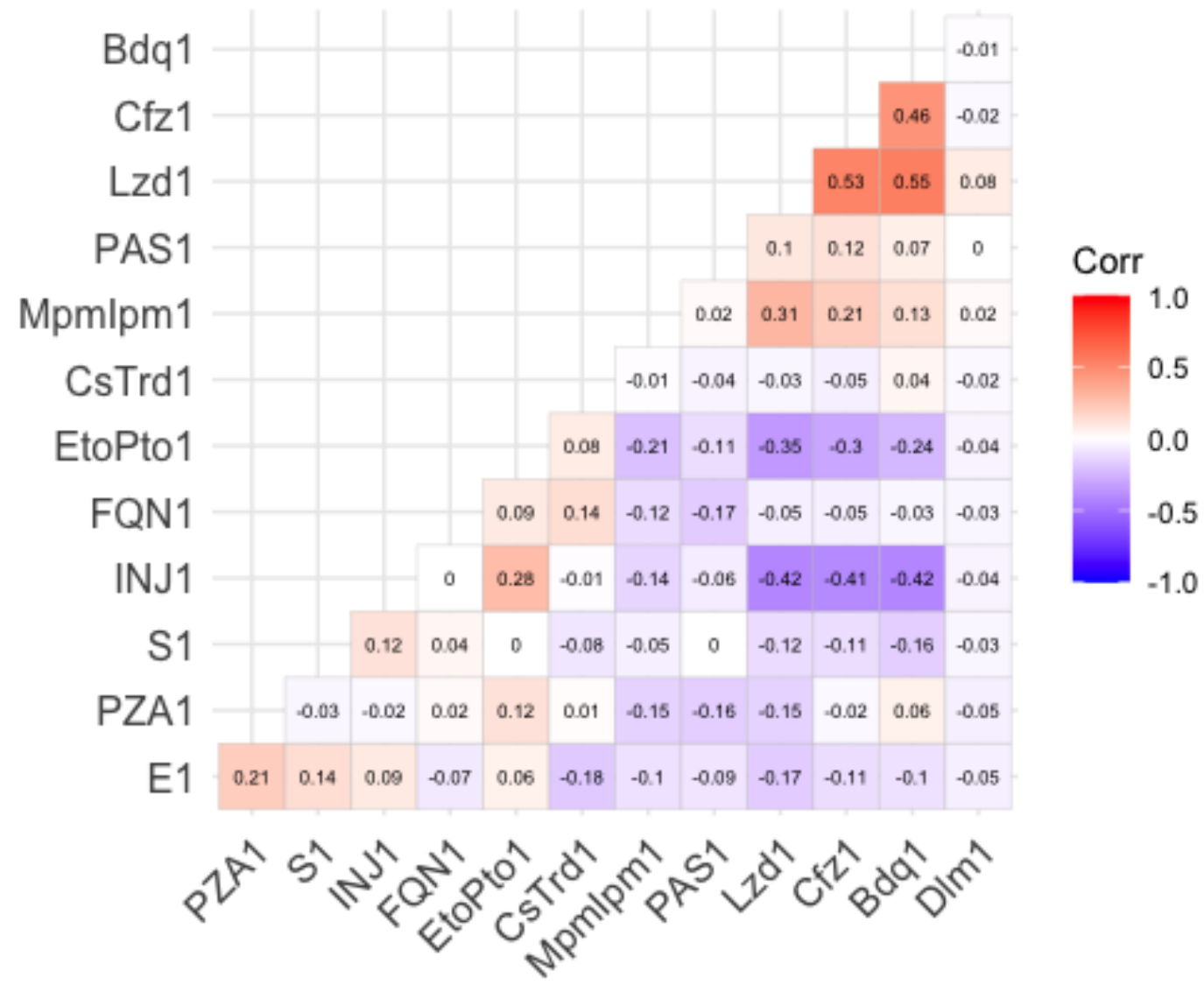

Supplementary Figure 2. Drug-use combination correlation.

E, Ethambutol. PZA, Pyrazinamide. S, Streptomycin. EtoPto, Ethionamide or Prothionamide. CsTrd, Cycloserine or Terizidone. Cfz, Clofazamine. Lzd, Linezolid. Bdq, Bedaquiline. Dlm, Delaminid. MpmIpm, Meropenem or Imipenem. FQN, fluoroquinolones. INJ, injectable drugs. SLI, second line injectables.

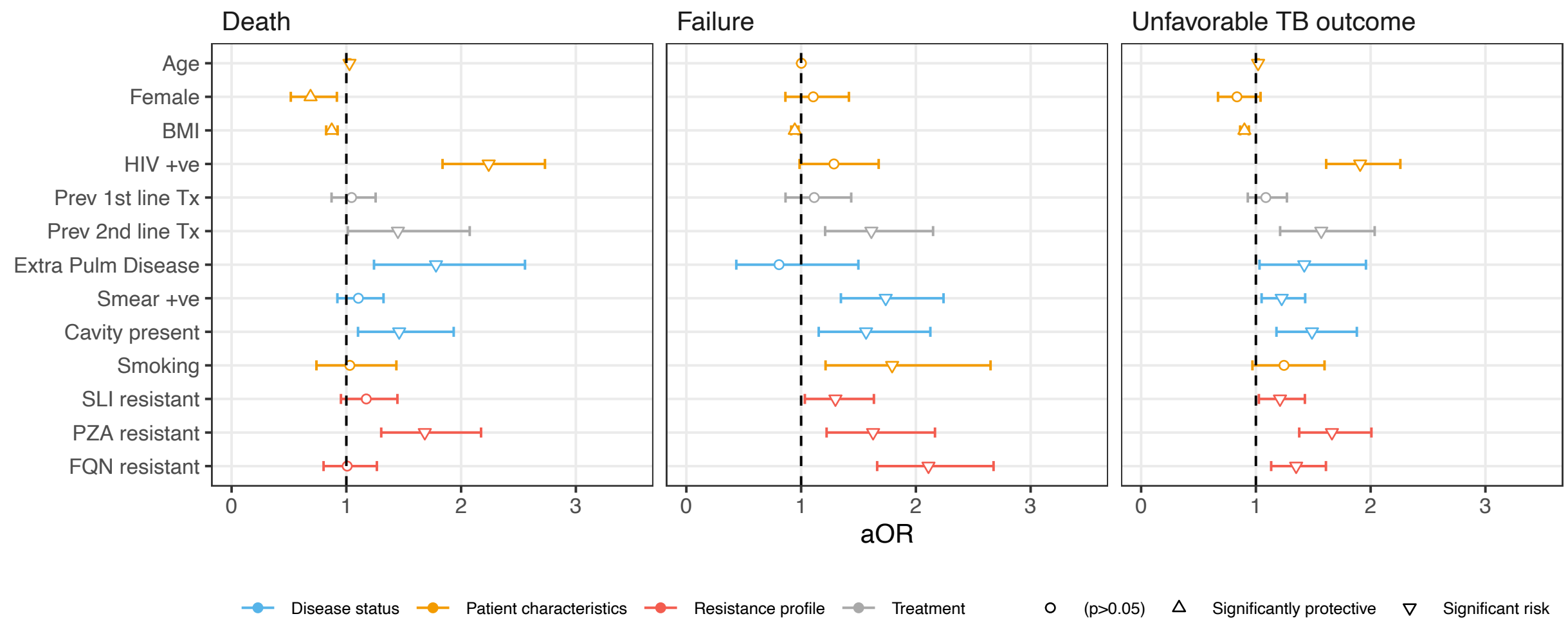

Supplementary Figure 3. Multivariable aORs for death, treatment failure and TB unfavorable outcome (death and treatment failure) of the clinical baseline predictors.

Models were built using mixed effects logistic regression, for Death (n=7122), Treatment failure (n=6497), and Unfavorable TB outcome (n=7750). Adjusted odds ratios (aOR) with 95% CIs are reported, with the center defined as the aOR and error bars representing the 95% CIs. Age and BMI were modeled as continuous variables; their aORs represent the change in odds of the outcome per one-year increase in age and per one-unit increase in BMI (kg/m^2), respectively; all other variables were modeled as categorical. Statistical significance was assessed using two-sided Wald tests derived from the mixed effects logistic regression models; estimates were pooled across imputed datasets following Rubin´s rules using the mice package; no adjustment for multiple comparisons was applied. HIV +ve: HIV positive. Prev 1st line Tx: Patient received past TB treatment with first line drugs. Prev 2nd line Tx: Patient received past TB treatment with second line drugs. Extra Pulm Disease: Extra pulmonary disease. Smear +ve: Smear positive. SLI: Second line injectables. PZA: Pyrazinamide. FQN: Fluoroquinolones.

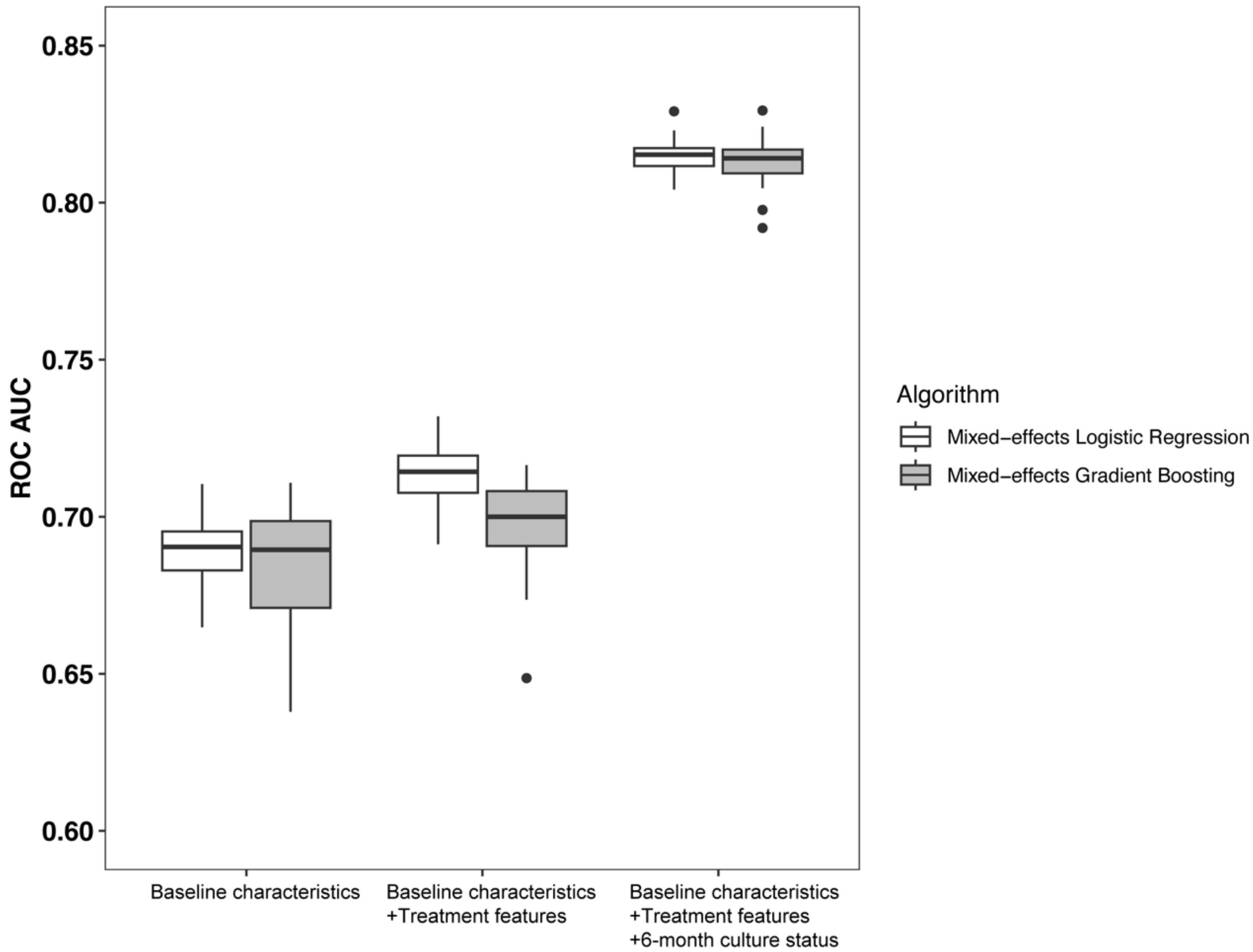

Supplementary Figure 4. The boxplot of ROC AUC of the mixed-effect logistic regression model and the mixed-effect gradient boosting model in the testing dataset (n=1629) among all 20 imputations.

Boxplots show the median; hinges indicate the 25th and 75th percentiles; whiskers extend to 1.5x the interquartile range (IQR). Outliers are represented by the single dots.

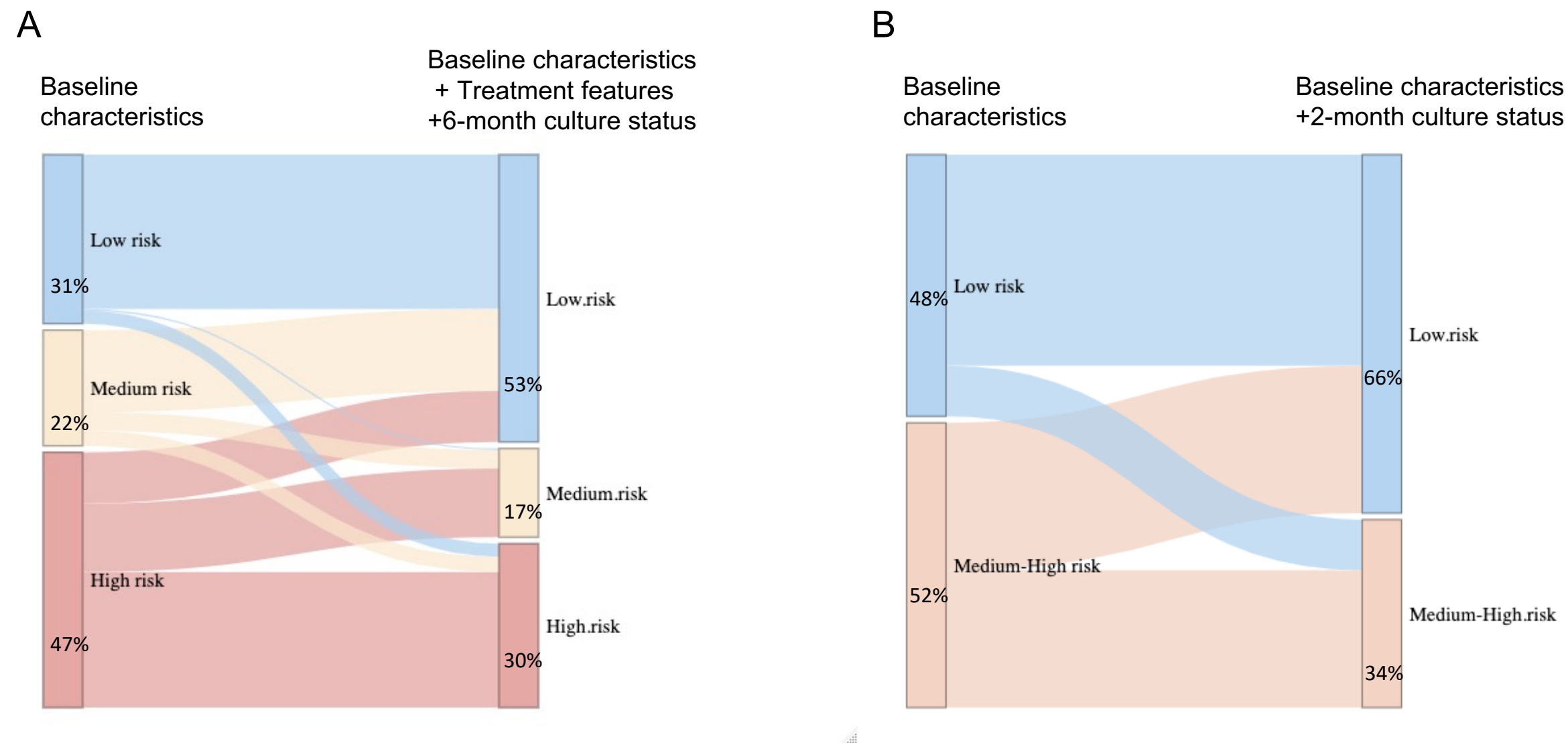

Supplementary Figure 5. Comparison of models on risk stratification re-evaluations.

A: The diagram shows how risk stratification changed (or unchanged) for MDT-TB patients in the analysis dataset from baseline (using Model A- Baseline characteristics), to 6 months after treatment categorization (using Model C- Baseline characteristics + Treatment features + 6-month culture status). Percentages indicate the proportion of patients in each risk strata for Model A and Model C overall, calculated from the 20 imputed datasets, based on the analysis dataset excluding patients who died or had treatment failure before 6 months of treatment (n=7109).

B: Risk stratification re-evaluation applied to MDT-TB patients in the validation dataset (n=378) from baseline (using Model A- Baseline characteristics), to 2 months after treatment categorization (using Model C- Baseline characteristics + 2-month culture status).

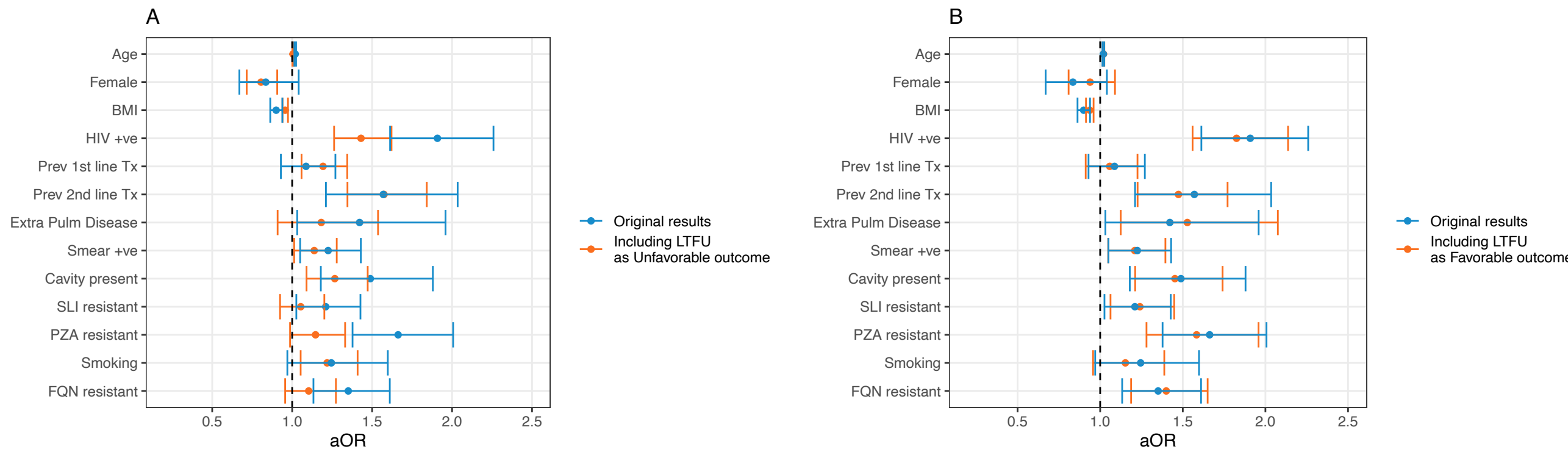

Supplementary Figure 6. Sensitivity analyses evaluating clinical predictors of TB unfavorable outcome with participants with a lost to follow up outcome considered either to have a successful or an unfavorable TB treatment outcome.

The plots display the associations (multivariable aOR) between clinical predictors and the probability of unfavorable TB outcome. The original results reported in the manuscript, which evaluated unfavorable TB outcome (failure or death) versus treatment success in the analysis dataset (N=7750) are represented in blue.

Panel A shows in red the results obtained when lost to follow-up patients (LTFU) (N=1375) were included in the analysis as an unfavorable outcome, evaluating death, failure or LTFU versus treatment success.

Panel B shows in red the results obtained when lost to follow-up patients (LTFU) (N=1375) were included in the analysis as a favorable outcome, evaluating death or failure versus treatment success or LTFU.

Adjusted odds ratios (aOR) with 95% CIs are reported, with the center defined as the aOR and error bars representing the 95% CIs. Age and BMI were modeled as continuous variables; their aORs represent the change in odds of the outcome per one-year increase in age and per one-unit increase in BMI (kg/m²), respectively; all other variables were modeled as categorical. Statistical significance was assessed using two-sided Wald tests derived from

the mixed effects logistic regression models; estimates were pooled across imputed datasets following Rubin´s rules using the mice package; no adjustment for multiple comparisons was applied. HIV +ve: HIV positive. Prev 1st line Tx: Patient received past TB treatment with first line drugs. Prev 2nd line Tx: Patient received past TB treatment with second line drugs. Extra Pulm Disease: Extra pulmonary disease. Smear +ve: Smear positive. SLI: Second line injectables. PZA: Pyrazinamide. FQN: Fluoroquinolones.

In general, the classification of lost to follow up as unfavorable TB outcome, reduced the strength and significance of the aORs (Supplementary Fig. 6A). On the other hand, categorizing the lost to follow-up patients as having a favorable TB outcome, reported similar results to the original analysis where these participants were excluded (Supplementary Fig. 6B).

These results suggested that participants with an outcome of LTFU were not comparable to those with treatment failure or death.

Consequently, participants with unknown outcome at the end of treatment were excluded from the main analysis.

Supplementary Table 1. ROC AUC performance of all models in the validation dataset during 5-fold cross-validation.

		Baseline characteristics	Baseline characteristics + Treatment features	Baseline characteristics + Treatment features + 6-month culture status
**Algorithm**	**Imputation dataset**	**Median [min - max]**	**Median [min - max]**	**Median [min - max]**
**Logistic Regression**	1	0.630 [0.604 - 0.897]	0.686 [0.635 - 0.923]	0.799 [0.772 - 0.970]
	2	0.671 [0.650 - 0.887]	0.683 [0.658 - 0.918]	0.808 [0.775 - 0.967]
	3	0.644 [0.631 - 0.894]	0.684 [0.646 - 0.923]	0.798 [0.773 - 0.969]
	4	0.656 [0.619 - 0.903]	0.693 [0.651 - 0.926]	0.808 [0.774 - 0.972]
	5	0.662 [0.625 - 0.897]	0.688 [0.667 - 0.922]	0.812 [0.781 - 0.969]
	6	0.669 [0.655 - 0.901]	0.700 [0.665 - 0.927]	0.816 [0.774 - 0.969]
	7	0.643 [0.639 - 0.888]	0.684 [0.650 - 0.916]	0.803 [0.778 - 0.967]
	8	0.658 [0.635 - 0.891]	0.683 [0.644 - 0.919]	0.789 [0.769 - 0.966]
	9	0.666 [0.652 - 0.899]	0.700 [0.663 - 0.924]	0.814 [0.782 - 0.970]
	10	0.656 [0.633 - 0.893]	0.681 [0.647 - 0.920]	0.812 [0.771 - 0.969]
	11	0.647 [0.612 - 0.891]	0.705 [0.644 - 0.923]	0.797 [0.770 - 0.968]
	12	0.647 [0.641 - 0.882]	0.691 [0.647 - 0.916]	0.807 [0.764 - 0.969]
	13	0.652 [0.640 - 0.893]	0.699 [0.652 - 0.923]	0.800 [0.776 - 0.969]
	14	0.658 [0.640 - 0.889]	0.687 [0.654 - 0.920]	0.809 [0.774 - 0.969]
	15	0.640 [0.606 - 0.886]	0.677 [0.636 - 0.918]	0.793 [0.768 - 0.969]
	16	0.642 [0.628 - 0.891]	0.679 [0.639 - 0.921]	0.803 [0.773 - 0.970]
	17	0.653 [0.631 - 0.890]	0.673 [0.643 - 0.919]	0.804 [0.776 - 0.968]
	18	0.687 [0.650 - 0.894]	0.700 [0.663 - 0.921]	0.812 [0.777 - 0.970]
	19	0.642 [0.590 - 0.897]	0.716 [0.644 - 0.921]	0.796 [0.770 - 0.969]
	20	0.655 [0.631 - 0.891]	0.689 [0.655 - 0.922]	0.806 [0.776 - 0.969]
		**Median (IQR)**	**Median (IQR)**	**Median (IQR)**
	**All**	**0.657 (0.641 - 0.687)**	**0.690 (0.667 - 0.792)**	**0.805 (0.780 - 0.846)**
**Gradient Boosting**	1	0.634 [0.575 - 0.858]	0.688 [0.626 - 0.890]	0.770 [0.763 - 0.963]
	2	0.674 [0.636 - 0.811]	0.701 [0.632 - 0.853]	0.788 [0.736 - 0.953]
	3	0.636 [0.608 - 0.837]	0.669 [0.612 - 0.881]	0.771 [0.764 - 0.954]
	4	0.642 [0.584 - 0.840]	0.670 [0.628 - 0.879]	0.782 [0.758 - 0.958]
	5	0.661 [0.619 - 0.840]	0.714 [0.613 - 0.876]	0.785 [0.744 - 0.955]
	6	0.668 [0.598 - 0.834]	0.701 [0.596 - 0.880]	0.790 [0.746 - 0.954]
	7	0.639 [0.619 - 0.814]	0.698 [0.622 - 0.859]	0.776 [0.732 - 0.947]
	8	0.643 [0.604 - 0.836]	0.678 [0.565 - 0.882]	0.770 [0.733 - 0.952]
	9	0.668 [0.645 - 0.836]	0.683 [0.630 - 0.863]	0.795 [0.750 - 0.955]
	10	0.654 [0.616 - 0.808]	0.679 [0.611 - 0.873]	0.782 [0.763 - 0.956]
	11	0.652 [0.640 - 0.801]	0.671 [0.617 - 0.866]	0.765 [0.749 - 0.960]
	12	0.662 [0.612 - 0.810]	0.685 [0.607 - 0.848]	0.782 [0.749 - 0.953]
	13	0.645 [0.620 - 0.809]	0.688 [0.611 - 0.876]	0.775 [0.748 - 0.954]
	14	0.675 [0.652 - 0.822]	0.694 [0.650 - 0.863]	0.785 [0.748 - 0.939]
	15	0.620 [0.586 - 0.790]	0.664 [0.617 - 0.847]	0.766 [0.737 - 0.954]
	16	0.658 [0.605 - 0.833]	0.679 [0.640 - 0.867]	0.777 [0.753 - 0.950]
	17	0.630 [0.602 - 0.797]	0.651 [0.611 - 0.862]	0.773 [0.749 - 0.952]
	18	0.672 [0.644 - 0.841]	0.686 [0.634 - 0.855]	0.796 [0.739 - 0.958]
	19	0.641 [0.559 - 0.826]	0.656 [0.637 - 0.869]	0.772 [0.736 - 0.956]
	20	0.658 [0.649 - 0.820]	0.693 [0.659 - 0.886]	0.796 [0.749 - 0.941]
		**Median (IQR)**	**Median (IQR)**	**Median (IQR)**
	**All**	**0.656 (0.634 - 0.685)**	**0.684 (0.649 - 0.730)**	**0.779 (0.763 - 0.821)**

IQR, Interquartile range.

Supplementary Table 2. Proportion of drug use by risk strata (using Model A- Baseline characteristics, n=7750).

	LOW RISK	MEDIUM RISK	HIGH RISK	TOTAL
E	48.0%	43.7%	45.7%	46.0%
PZA	81.0%	79.9%	82.5%	81.4%
S	12.2%	9.2%	5.7%	8.5%
SLI	83.6%	84.1%	78.8%	81.4%
CfxOfx	28.5%	28.4%	19.6%	24.3%
MfxLfxGfx	72.7%	71.8%	72.1%	72.2%
EtoPto	80.3%	80.9%	77.7%	79.2%
CsTrd	84.1%	88.2%	84.1%	85.0%
PAS	38.7%	39.4%	45.7%	42.2%
Lzd	12.7%	16.4%	25.5%	19.5%
Cfz	8.4%	10.9%	16.7%	12.9%
MpmIpm	1.6%	2.6%	3.6%	2.8%
Bdq	10.7%	18.1%	30.9%	21.8%
Dlm	0.7%	1%	0.9%	0.9%

E, Ethambutol. PZA, Pyrazinamide. S, Streptomycin. SLI, second line injectables. CfxOfx Ciprofloxacin or Ofloxacin. MfxLfxGfx, Moxifloxacin, Levofloxacin or Gatifloxacin. EtoPto, Ethionamide or Prothionamide. CsTrd, Cycloserine or Terizidone. Lzd, Linezolid. Cfz, Clofazamine. MpmIpm, Meropenem or Imipenem. Bdq, Bedaquiline. Dlm, Delaminid.

Supplementary Table 3. Treatment outcomes definitions for MDR/RR-TB patients

Outcome	WHO definition[a]	Laserson and colleagues definition[b]
Failure	Treatment terminated or need for permanent regimen change of at least two anti-TB drugs because of: lack of conversion by the end of the intensive phase, or bacteriological reversion in the continuation phase after conversion to negative, or evidence of additional acquired resistance to fluoroquinolones or second-line injectable drugs, or adverse drug reactions (ADRs).	Treatment will be considerd failure if two or more of the five cultures recorded in the final 12 months are positive, or if any one of the final three cultures is positive, or if a clinical decision has been made to terminate treatment early due to poor response or adverse events.
Death	Death for any reason during the course of treatment	Death for any reason during the course of treatment
Cure	Treatment completed as recommended by the national policy without evidence of failure AND three or more consecutive cultures taken at least 30 days apart are negative after the intensive phase.	Treatment completed according to country protocol and consistently culture-negative (with at least five results) for the final 12 months of treatment.
Completion	Treatment completed as recommended by the national policy without evidence of failure BUT no record that three or more consecutive cultures taken at least 30 days apart are negative after the intensive phase.	Treatment completed according to country protocol but does not meet the definition for cure or treatment failure due to lack of

[a]Reproduced from "WHO. Definitions and reporting framework for tuberculosis—2013 revision. Geneva: World Health Organization,2013. A.2.2 Outcomes for RR-TB/MDR-TB/XDR-TB patients treated using second-line treatment"

[b]Reproduced from "Laserson KF, Thorpe LE, Leimane V, et al. Speaking the same language: treatment outcome definitions for multidrug-resistant tuberculosis. Int J Tuberc Lung Dis 2005; 9: 640–45. Treatment outcome definitions for MDR-TB patients"

Supplementary Table 4. Six-Months culture conversion and 2-Months culture conversion coefficients comparison

	**Analysis Dataset Model C**	**Validation Dataset Model C**	**Validation Dataset Univariate**	**Validation Dataset Model C Refit all**
Predictor coefficient	Estimate	Estimate	Estimate	Estimate
Intercept	0.70	-1.53	-1.1	-1.32
**6m culture conv/ 2m culture conv**	**-2.41**	**-2.16**	**-2.28**	**-2.37**
Age	0.02	0.02	--	0.07
Female	-0.22	-0.22	--	-1.64
BMI	-0.11	-0.11	--	-0.11
HIV +ve	0.64	0.64	--	0.3
SLI resistant	0.30	0.30	--	0.6
Smear +ve	0.14	0.14	--	1.25
Cavity present	0.31	0.31	--	0.24
Smoking	0.26	0.26	--	-1.45
PZA resistant	0.28	0.28	--	0.28
FQN resistant	0.25	0.25	--	-1

HIV +ve: HIV positive. Prev 1st line Tx: Patient received past TB treatment with first line drugs. Prev 2nd line Tx: Patient received past TB treatment with second line drugs. Extra Pulm Disease: Extra pulmonary disease. Smear +ve: Smear positive. SLI: Second line injectables. PZA: Pyrazinamide. FQN: Fluoroquinolones. 6m culture conv: Six-month culture conversion. Age and BMI were modeled as continuous variables.
