# Supplementary material for: Risk stratification for MDR/RR-TB: a comprehensive integrated patient-level analysis from TB programs and contemporary clinical trials
Source: Nat Commun. 2026 Sep 22;17:11337. doi: 10.1038/s41467-026-77931-0 (PMC13728123; doi:10.1038/s41467-026-77931-0)
Supplement: Supplementary file 2 [file 41467_2026_77931_MOESM2_ESM.pdf]

nature portfolio

Corresponding author(s): Radojka M. Savic

Last updated by author(s): Aug 26, 2026

Reporting Summary

Nature Portfolio wishes to improve the reproducibility of the work that we publish. This form provides structure for consistency and transparency in reporting. For further information on Nature Portfolio policies, see our Editorial Policies and the Editorial Policy Checklist.

Statistics

For all statistical analyses, confirm that the following items are present in the figure legend, table legend, main text, or Methods section.

n/a	Confirmed	
☐	☒	The exact sample size (*n*) for each experimental group/condition, given as a discrete number and unit of measurement
☒	☐	A statement on whether measurements were taken from distinct samples or whether the same sample was measured repeatedly
☐	☒	The statistical test(s) used AND whether they are one- or two-sided<br>*Only common tests should be described solely by name; describe more complex techniques in the Methods section.*
☐	☒	A description of all covariates tested
☐	☒	A description of any assumptions or corrections, such as tests of normality and adjustment for multiple comparisons
☐	☒	A full description of the statistical parameters including central tendency (e.g. means) or other basic estimates (e.g. regression coefficient) AND variation (e.g. standard deviation) or associated estimates of uncertainty (e.g. confidence intervals)
☐	☒	For null hypothesis testing, the test statistic (e.g. *F*, *t*, *r*) with confidence intervals, effect sizes, degrees of freedom and *P* value noted<br>*Give P values as exact values whenever suitable.*
☒	☐	For Bayesian analysis, information on the choice of priors and Markov chain Monte Carlo settings
☐	☒	For hierarchical and complex designs, identification of the appropriate level for tests and full reporting of outcomes
☐	☒	Estimates of effect sizes (e.g. Cohen's *d*, Pearson's *r*), indicating how they were calculated

Our web collection on statistics for biologists contains articles on many of the points above.

Software and code

Policy information about availability of computer code

Data collection	Data collection and formatting was performed using the R statistical software version 3.6.2
Data analysis	All data analysis was performed using the R statistical software version 3.6.2

For manuscripts utilizing custom algorithms or software that are central to the research but not yet described in published literature, software must be made available to editors and reviewers. We strongly encourage code deposition in a community repository (e.g. GitHub). See the Nature Portfolio guidelines for submitting code & software for further information.

Data

Policy information about availability of data

All manuscripts must include a data availability statement. This statement should provide the following information, where applicable:

- Accession codes, unique identifiers, or web links for publicly available datasets
- A description of any restrictions on data availability
- For clinical datasets or third party data, please ensure that the statement adheres to our policy

The individual patient data used in this study were part of the WHO TB treatment individual patient data (TB-IPD) platform, a global data repository jointly established by the World Health Organization Global TB Programme and University College London (UCL). Access to these data is subject to approval by the Data Access Committee (see https://www.ucl.ac.uk/global-health/research/z-research/tb-ipd-platform for further details). Data from the Phase 3 trials used for external validation (Nix-TB, ZeNix, and TB-PRACTECAL) are available from the respective trial sponsors, subject to their data-sharing policies.

Research involving human participants, their data, or biological material

Policy information about studies with human participants or human data. See also policy information about sex, gender (identity/presentation), and sexual orientation and race, ethnicity and racism.

Reporting on sex and gender	Sex information was available and included in the analysis. In the analysis dataset, 59.6% of the participants were male, and 61% of the participants were male in the validation dataset. Sex was accounted and evaluated as predictor of outcome in the analysis.
Reporting on race, ethnicity, or other socially relevant groupings	The analysis does not report on race, ethnicity or other socially relevant grouping. Each model was adjusted for study and center using random intercepts.
Population characteristics	Population characteristics of the analysis dataset are available in Table 1 of the manuscript. In summary, the mean age was 37.6 years, mean body mass index (BMI) was 20.23 kg/m2, 59.6% were male, and 34.4% were living with HIV. Baseline characteristics of participants did not differ across the analysis and validation datasets, with participants in the validation dataset having a mean age of 36 years, mean BMI of 21 kg/m2, 61% were male and 32% HIV positive.
Recruitment	Recruitment was performed in the parent trials that were previously published,
Ethics oversight	Assembly and use of this individual patient data was approved by the Research Institute of the McGill University Health Centre as well as by participating sites, as necessary.The protocol for each study (for both the analysis and validation datasets) was reviewed and approved by ethics committees and regulatory committees described in the original publications, and all participants provided written informed consent.

Note that full information on the approval of the study protocol must also be provided in the manuscript.

Field-specific reporting

Please select the one below that is the best fit for your research. If you are not sure, read the appropriate sections before making your selection.

☒ Life sciences ☐ Behavioural & social sciences ☐ Ecological, evolutionary & environmental sciences

For a reference copy of the document with all sections, see nature.com/documents/nr-reporting-summary-flat.pdf

Life sciences study design

All studies must disclose on these points even when the disclosure is negative.

Sample size	No sample size calculation was performed. this was a pooled individual patient-data analysis that used all available data from 52 published observational and experimental studies of MDR-TB treatment performed across 38 countries and collected between 2009 abd 2018. Likewise, all available data coming from the studies used for validation (Nix-TB (NCT02333799) , ZeNix (NCT03086486) , and TB-PRACTECAL (NCT02589782)) was also included
Data exclusions	Data exclusions are listed in figure 1. In summary, participants who were lost to follow up, with missing data on treatment duration, and missing time to culture conversion were excluded from analysis.
Replication	The code for the analysis, that was performed in the R statistical software, is annotated for reproducibility and available upon request..
Randomization	Randomization and blinding was performed in the parent trials that were previously published.
Blinding	Randomization and blinding was performed in the parent trials that were previously published.

Reporting for specific materials, systems and methods

We require information from authors about some types of materials, experimental systems and methods used in many studies. Here, indicate whether each material, system or method listed is relevant to your study. If you are not sure if a list item applies to your research, read the appropriate section before selecting a response.

Materials & experimental systems

n/a	Involved in the study
☒	☐ Antibodies
☒	☐ Eukaryotic cell lines
☒	☐ Palaeontology and archaeology
☒	☐ Animals and other organisms
☐	☒ Clinical data
☒	☐ Dual use research of concern
☒	☐ Plants

Methods

n/a	Involved in the study
☒	☐ ChIP-seq
☒	☐ Flow cytometry
☒	☐ MRI-based neuroimaging

Clinical data

Policy information about clinical studies

All manuscripts should comply with the ICMJE guidelines for publication of clinical research and a completed CONSORT checklist must be included with all submissions.

Clinical trial registration	This analysis included individual level data from 12,938 patients with pulmonary TB and confirmed rifampin resistance from 52 observational and experimental studies of MDR/RR-TB treatment performed across 38 countries/regions and collected between 2009 and 2018, which metanalysis was already published (Ahmad, N. et al. Treatment correlates of successful outcomes in pulmonary multidrug-resistant tuberculosis: an individual patient data meta-analysis. The Lancet 392, 821–834 (2018)), with no overlap with the results presented here. The validation dataset included data from three Phase 3 trials, Nix-TB (NCT02333799) performed between 2015 and 2019, ZeNix (NCT03086486) performed between 2017 and 2021, and TB-PRACTECAL (NCT02589782) performed between 2017 and 2022.
Study protocol	The protocol for each study was reviewed and approved by ethics committees and regulatory committees described in the original publications
Data collection	Data acquisition, assembly and use of the individual patient data was performed by the Research Institute of the McGill University Health Centre as well as by participating sites, as necessary. Data was collected between 2009 and 2018. For the validation dataset, data acquisition, assembly and use of the individual data was approved and coordinated by TBAlliance and Médecins Sans Frontières, as well as participating sites if necessary.
Outcomes	The primary efficacy endpoint was unfavorable TB outcome (defined as treatment failure or death), compared to treatment success (defined as cure or completion). Each efficacy endpoint (any unfavorable TB outcome, death or failure was analyzed using mixed-effect logistic regression models to identify predictors of each endpoint.

Plants

Seed stocks	*Report on the source of all seed stocks or other plant material used. If applicable, state the seed stock centre and catalogue number. If plant specimens were collected from the field, describe the collection location, date and sampling procedures.*
Novel plant genotypes	*Describe the methods by which all novel plant genotypes were produced. This includes those generated by transgenic approaches, gene editing, chemical/radiation-based mutagenesis and hybridization. For transgenic lines, describe the transformation method, the number of independent lines analyzed and the generation upon which experiments were performed. For gene-edited lines, describe the editor used, the endogenous sequence targeted for editing, the targeting guide RNA sequence (if applicable) and how the editor was applied.*
Authentication	*Describe any authentication procedures for each seed stock used or novel genotype generated. Describe any experiments used to assess the effect of a mutation and, where applicable, how potential secondary effects (e.g. second site T-DNA insertions, mosiacism, off-target gene editing) were examined.*
