# Supplementary material for: Risk stratification for MDR/RR-TB: a comprehensive integrated patient-level analysis from TB programs and contemporary clinical trials
Source: Nat Commun. 2026 Sep 22;17:11337. doi: 10.1038/s41467-026-77931-0 (PMC13728123; doi:10.1038/s41467-026-77931-0)
Supplement: Supplementary file 3 [file 41467_2026_77931_MOESM3_ESM.pdf]

nature portfolio

Peer Review File

Risk Stratification for MDR/RR-TB: A Comprehensive Integrated Patient-Level Analysis from TB Programs and Contemporary Clinical Trials

Corresponding Author: Dr Rada Savic

This manuscript has been previously reviewed at another journal. This document only contains information relating to versions considered at Nature Communications.

This file contains all reviewer reports in order by version, followed by all author rebuttals in order by version.

Version 0:

Reviewer comments:

Reviewer #5

(Remarks to the Author)
See attachment.

Open Access This Peer Review File is licensed under a Creative Commons Attribution 4.0 International License, which permits use, sharing, adaptation, distribution and reproduction in any medium or format, as long as you give appropriate credit to the original author(s) and the source, provide a link to the Creative Commons license, and indicate if changes were made.
In cases where reviewers are anonymous, credit should be given to 'Anonymous Referee' and the source.
The images or other third party material in this Peer Review File are included in the article's Creative Commons license, unless indicated otherwise in a credit line to the material. If material is not included in the article's Creative Commons

license and your intended use is not permitted by statutory regulation or exceeds the permitted use, you will need to obtain permission directly from the copyright holder.
To view a copy of this license, visit https://creativecommons.org/licenses/by/4.0/

RESPONSES TO THE REVIEWER

We would like to thank the reviewer for the time and effort dedicated to evaluating our manuscript. We have addressed all comments and suggestions and revised the manuscript accordingly. Below, we provide a point-by-point response to each comment.

The authors have addressed my questions. The added mixed effects boosted regression tree model (BRT) is a plus, although it is a little surprising to me that it is no better than the all linear effects logistic model. One possible reason is that all predictors are dichotomized for both the BRT and the logistic model. BRT takes full advantage of continuous covariates. I'd suggest keeping variables like age and BMI as continuous.
Thank you for this thoughtful suggestion. Following the reviewer´s suggestion, we have included age and BMI as continuous variables in both the logistic regression and the boosted regression tree (BRT) models. As expected, model discrimination improved slightly for both approaches. For example, in the model that included baseline characteristics, treatment features and six month culture status, the AUROC increased from 0.79 to 0.82 for the logistic regression model (3.5% relative increase) and from 0.78 to 0.81 for the XGBoost model (4.8% relative increase). Notably, the logistic regression model continued to perform similarly to, or slightly better than the BRT model across all predictor sets, with substantial overlap in the cross-validation ROC AUC distributions (Supplementary Figure S4, attached), and was therefore selected for risk stratification. We have updated the manuscript (Figures, Tables, Results) accordingly, with age and BMI now modelled as continuous covariates.

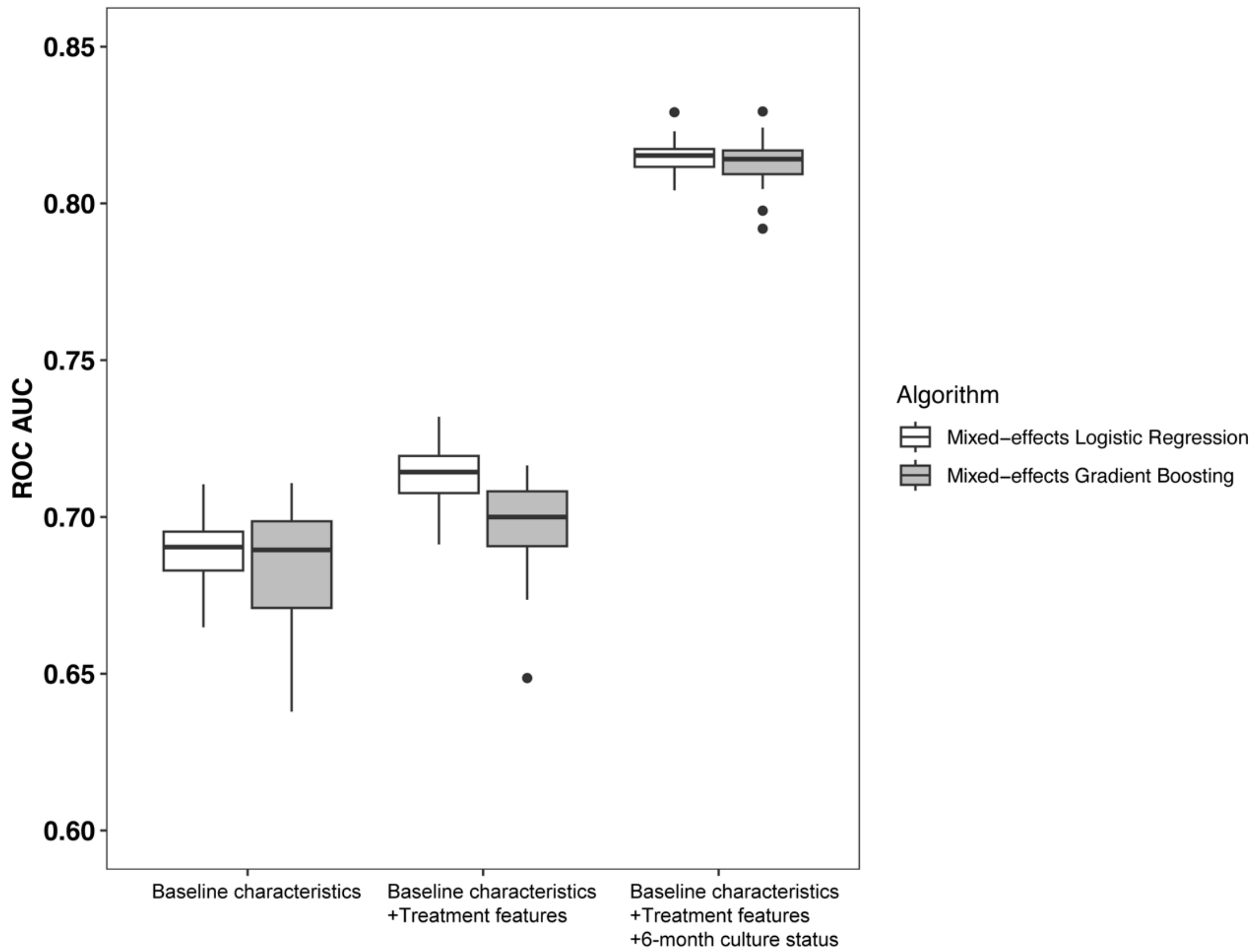

Figure S4. The boxplot of ROC AUC of the mixed-effect logistic regression model and the mixed-effect gradient boosting model in the testing dataset among all 20 imputations.

I have a few additional minor comments:

1. Line 52, “The 6-month BPaLM regimen … is preferred in programmatic conditions over the previous shorter (9-month) regimen” sounds like 9-mon is shorter than 6-mon.
   Thank you for pointing this out. We have revised the sentence to remove the ambiguity and now it reads:

   The 6-month BPaLM regimen … is preferred in programmatic conditions over the 9-month regimen

2. While I understand the authors’ reasons, I still feel a bit uneasy about using 2-mon conversion as a surrogate for 6-mon conversion when they applied the model to the validation dataset. I’m not a TB expert, but I feel claiming 2-mon culture conversion is a good surrogate for 6-mon conversion is a bit stretch, unless there are references backing it up. However, I cannot think of a better way, so I will leave this question to the readers, because I don’t think this issue should hamper its publication. However, I do suggest adding a column in Table S4 to show the coefficients of a fully refitted model (i.e., not forcing all other coefficient to be the same as the primary model as in the second column).
   Thank you. We have added the suggested column in Table S4.

3. Line 92, “77% percent of patients in the analysis dataset were ever treated with a drug for which they had confirmed resistance” is a bit confusing as “ever treated” could mean before the baseline. May be “were ever treated for MDR/RR-TB with…”?
   We have revised the text as suggested.

4. Line 103, “unfavorable outcome” in the section subtitle may be misleading as no such result is presented in this section.
   We agree with the reviewer and have removed unfavorable outcome from the section title.

5. Clarify aOR, ROC and AUC results presented in Fig. 3, Fig. S3, and lines 104-115, 124-139 were based on the whole analysis dataset and accounted for missing values. Split and cross-validation were only done for comparing BRT and logistic regression (if my understanding is correct). My understanding is based on the caption of Fig 3, line 557-558, “ROC curve plots for the three models developed using all data”.
   Thank you for this comment. Your understanding is correct.
   The aORs, ROC curves, and AUCs presented in Fig. 3, Fig. S3, and the corresponding Results sections were obtained from models fitted using the full analysis dataset after accounting for missing values. The train/test split and cross-validation procedures were used exclusively for the comparison of predictive performance between the boosted regression tree and logistic regression models(also accounting for missing values). We have included further clarification in the Methods to avoid ambiguity:

   Train/test splitting and cross-validation were performed only for the comparison of predictive performance between the boosted regression tree and logistic regression models. The model with better performance was selected to fit the full analysis dataset as final models.

6. In the captions of Fig 3 and Fig 4, avoid using A, B, C to label both models and subplots.
   Thank you. We have removed the A, B, C labels from the models and subplots.

7. Lines 143, 150-152, are the p-values from log-rank test? 97%, 90% and 85% seem to be the proportions of no unfavorable outcome at the end of month 28; so I'm not sure the p-values are comparing these final probabilities or comparing the survival curves using the log-rand test.
   We thank the reviewer for pointing out this ambiguity. We confirm that the p-values referred to the log-rank test comparing the overall probability of treatment success over the entire follow-up period between risk phenotypes (Fig. 4C), and not the proportions at the end of treatment. We have corrected the text accordingly, specifying the log-rank test, and now report the end-of-treatment proportions descriptively, without an accompanying statistical comparison. The text now reads as follows:

   The proportion of patients with no unfavorable outcome over time since treatment initiation differed between low-risk and medium and high-risk phenotypes (Model A, p=0.0059; Model C, p=0.0012; log-rank test) (Fig. 4C). At the end of treatment, the proportion of treatment success was 97% with Model A and 97% with Model C in the low-risk phenotype, and 91% with Model A and 88% with Model C in the medium and high-risk phenotypes (Fig. 4C).

8. Lines 162-163, these percents seem contradicting the model-estimated aOR that LZD and BDQ were associated with reduced risk of unfavorable outcomes as shown in Figure 3A.
   Thank you for this important observation. The percentages reported in lines 162–163 are unadjusted descriptive. Based on the data, linezolid (LZD) and bedaquiline (BDQ) are more likely to be prescribed to patients with more severe or complicated disease (patients categorized as high risk at baseline), resulting in a higher crude frequency among individuals with unfavorable outcomes. In contrast, the adjusted odds ratios shown in Figure 3 account for these differences and indicate an independent protective association of both drugs with unfavorable outcomes. We have clarified this distinction in the Results to avoid confusion.

9. Fig S5, caption has redundancy (lines 59-62).
   Thank you for pointing this out. We have revised the caption accordingly to remove the redundancy.
